# Cytotoxic and Cytolytic Cnidarian Venoms. A Review on Health Implications and Possible Therapeutic Applications

**DOI:** 10.3390/toxins6010108

**Published:** 2013-12-27

**Authors:** Gian Luigi Mariottini, Luigi Pane

**Affiliations:** Department of Earth, Environment and Life Sciences, University of Genova, Viale Benedetto XV 5, Genova I-16132, Italy; E-Mail: pane@unige.it

**Keywords:** Cnidaria, venom, cytotoxicity, cell cultures

## Abstract

The toxicity of Cnidaria is a subject of concern for its influence on human activities and public health. During the last decades, the mechanisms of cell injury caused by cnidarian venoms have been studied utilizing extracts from several Cnidaria that have been tested in order to evaluate some fundamental parameters, such as the activity on cell survival, functioning and metabolism, and to improve the knowledge about the mechanisms of action of these compounds. In agreement with the modern tendency aimed to avoid the utilization of living animals in the experiments and to substitute them with *in vitro* systems, established cell lines or primary cultures have been employed to test cnidarian extracts or derivatives. Several cnidarian venoms have been found to have cytotoxic properties and have been also shown to cause hemolytic effects. Some studied substances have been shown to affect tumour cells and microorganisms, so making cnidarian extracts particularly interesting for their possible therapeutic employment. The review aims to emphasize the up-to-date knowledge about this subject taking in consideration the importance of such venoms in human pathology, the health implications and the possible therapeutic application of these natural compounds.

## 1. Introduction

In natural environments a lot of toxic substances are produced by organisms for defense/offence purposes. These compounds can have an impact on ecosystem functioning, on competition among species, as well as on some human activities and public health; nevertheless, in spite of this, several of them were seen to have potentially useful pharmacological properties. In particular, in aquatic environments, the biodiversity and the associated chemical diversity can be a practically unlimited source of new bioactive substances useful in developing new drugs [1]. 

In this framework, the interest around marine venoms has increased during the last three to four decades, even though to date their mechanism of action is still largely unknown and under debate [2]. 

Cnidarians are responsible for envenomations occurring during some human activities carried out in the marine environment, both in work situations, such as fishing, and recreational ones, such as bathing; these problems involve the management of public health and are especially connected to jellyfish outbreaks occurring in coastal marine ecosystems on a global scale [3]. Cnidarians are well known producers of complex mixtures of proteinaceous venoms used for defence as well as for prey capture [4], contained in capsules of protein nature—the nematocysts, which are secretory products of the Golgi apparatus synthesized by high specialized cells called nematocytes [5]. The capsule contains a tightly wrapped and spiralized thread which is extruded under adequate physico-chemical stimuli, injecting the venom in the prey or in the attacker. Cnidarian stinging can induce local and systemic symptoms and pose a serious threat to human health along Asian and Australian coasts as well as in tropical oceanic waters where extremely venomous jellyfish and anemones able to induce severe and also lethal envenomations are common. The damage induced by cnidarian venoms has been essentially ascribed to a pore formation mechanism or to oxidative stress [2]. 

In spite of their toxicity, Cnidaria have long been indicated as a potential source of natural bioactive compounds of pharmacological concern useful to develop new drugs or biomedical materials [6]. Some bioactive substances, such as prostaglandins (15R)-PGA2 from the gorgonian *Plaxaura homomalla* [7], the Palytoxin local anaesthetic and vasoconstrictive agent from the zoanthid *Palythoa toxica* [8], Pseudopterosin [9], Sarcodictyns and Eleutherobin have been discovered in these organisms. Hence, during recent decades, the interest for the biology and utilization of cnidarians has grown and a number of metabolites, anticancer and antioxidant compounds have been isolated in the interest of human health [3], and have been seen to have activity at the cellular level, making them a possible source of new drugs. Therefore, taking into account the modern tendency to utilize cultured cells in the research with the view to lower the need for *in vivo* experimentation, the aim of this paper is to review the up-to-date knowledge about the *in vitro* cytotoxicity of cnidarian venoms emphasizing their mechanisms of action and their possible therapeutic application against neurologic, haematologic, infectivologic and oncologic diseases, as well as their hemolytic properties.

## 2. Hemolytic Effects of Cnidarian Venoms

The hemolytic effects of some cnidarian venoms are long known [6]. During the second half of the last Century hemolysins have been recognized in the box jellyfish [10,11,12] in the Portuguese Man-of-War [13], in sea anemones [14,15,16,17,18], and in other Cnidaria [19,20]; the role of phospholipases in the hemolytic activity of cnidarian venoms was also emphasized [21]. To date, the research on the hemolytic effects of Cnidaria is focused mainly on Anthozoans (sea anemones, soft corals), Scyphozoans and Cubozoans and several species are known to be responsible for the cytolytic effects on different mammalian red blood cells (RBC). Other species have been considered in a recent paper [22] that concened the hemolysis induced in sheep RBC after treatment with extracts from the anthomedusan *Pandea rubra*, the trachimedusae *Arctapodema* sp., *Colobonema sericeum*, *Crossota rufobrunnea*, *Halicreas minimum* and *Pantachogon haeckeli*, the narcomedusae *Aeginura grimaldii*, *Aegina citrea* and *Solmissus* sp. and the scyphozoan Coronatae *Atolla vanhoeffeni*, *Atolla wyvillei* and *Periphylla periphylla*; only extracts from *Arctapodema* sp., *Colobonema sericeum*, and *Crossota rufobrunnea* were reported to be actively cytolytic with ED_50_ values of 110, 190 and 100 mg/mL, respectively.

### 2.1. Hemolytic Sea Anemone (Anthozoa) Venoms

In a comprehensive review, Anderluh and Maček [23] indicated that “more than 32 species of sea anemones have been reported to produce lethal cytolytic peptides and proteins” and classified the cytolysins into four polypeptide groups: I (5–8 kDa peptides) that are able to make pores in membranes containing phosphatidylcholine; II (20 kDa actinoporins) that typically associate with membranes containing sphingomyelin making cation-selective pores; III that includes lethal 30–40 kDa cytolytic phospholipases A2; IV including only metridiolysin from *Metridium senile* (80 kDa), a thiol-activated cytolysin inhibited by cholesterol or phosphatides [23]. 

In the late 1980s, a hemolytic toxin acting at the membrane level and having phospholipase activity was isolated from the sea anemone *Stoichactis helianthus*; its conjugate with an antibody towards IOR-T6 antigen expressed on immature T lymphocytes was tested as a potential anti-cancer agent. The hybrid molecule (IOR-T6-HT) did not exhibit hemolytic activity unless it was reduced, but was toxic for cells (CEM) expressing the IOR-T6 antigen and non-toxic for cells (K562) not bearing the antigen [24]. Subsequently, the basic protein UpI isolated from crude extract from *Urticina piscivora* was found to be hemolytic at concentrations as low as 10^−10^ M on rat, guinea pig, dog, pig and human RBC; this result was confirmed also through scanning electron microscopy observations that evidenced structural damage to rat and guinea pig RBC membranes. Sphingomyelin but not cholesterol was able to inhibit hemolytic effects in a concentration-dependent manner [25].

The importance of the *N*-terminal amphiphilic α-helix for the functionality of actinoporins was reported for the recombinant hemolytic Src I from the sea anemone *Sagartia rosea*; in fact, notwithstanding Src I was found to be strongly hemolytic (50% red blood cells lysed at a concentration of 0.43 μg/mL), the occurrence of an additional peptide at the *N*-terminus of Src I decreases the hemolytic activity of the fusion protein Trx-Src I, because the *N*-terminal α-helix of cytolysins, being strongly amphipathic, interacts with lipid membranes [26].

Recent results indicate that the crude venom from the sea anemone *Aiptasia mutabilis* has dose-response hemolytic effects against human erythrocytes probably due to a pore-forming mechanism that can be prevented by Ca^2+^, Ba^2+^ and Cu^2+^, papain and polyethylenglycole and to a minor extent by Mg^2+^ and K^+^ treatment [27].

### 2.2. Hemolytic Octocoral (Anthozoa) Venoms

Eunicellin-type diterpenoids Litophynols A and B, litophynins E and H, and I monoacetate from the mucus of the soft coral *Litophyton* sp. (Alcyonacea) were found to have hemolytic properties on a 2% rabbit erythrocyte suspension [28]. Recently a hemolytic toxin was identified in the soft coral *Sarcophyton trocheliophorum* (Alcyonacea); the crude extract was highly cytotoxic (EC_50_ = 50 ng/mL) against human erythrocytes and haemolytic, with a halo of 12 mm caused by 50 μg of protein. The hemolysis was observed to be increased in condition of alkaline and neutral pH and reduced at acidic pH; furthermore, hemolysis is reduced after toxin treatment with freezing-thawing cycles [29]. A modified steroid (18-acetoxipregna-1,4,20-trien-3-one) isolated from *Carijoa riisei* was shown to be not hemolytic at 12.5 μg/mL and slightly hemolytic (2.3% and 6%) at 25 μg/mL and 50 μg/mL, respectively [30].

### 2.3. Hemolytic Cubozoan Venoms

The hemolytic properties of cubozoans are long known [10]. In a comprehensive review about cubozoan cytolysins, Brinkman and Burnell [31] reported that three highly toxic lethal and hemolytic proteins (CrTX-I, CrTX-II and CrTX-III) were isolated from tentacle extracts of *Carybdea rastoni* and described; these proteins induced platelet aggregation, acted as calcium-dependent vasoconstrictors and damaged the uptake/storage mechanisms of noradrenaline [32,33,34]. In the 1990s haemolytic factors were partially purified from *Carybdea rastoni* [35] and furthermore a 107 kDa cytolysin from *Carybdea marsupialis*—CARTOX—lacking phospholipase C activity and acting as a pore-forming protein was found to be hemolytic to sheep erythrocytes [36]. From specimens collected in the Caribbean Sea, three cytolysins (220, 139 and 36 kDa) hemolytic to human erythrocytes were recently isolated [37]. 

The hemolytic properties of *Carybdea alata* venom were attributed to a protein (CAH1) with an apparent mass of 42 kDa which was subsequently purified studying sheep RBCs [38]; the same research group supposed that the hemolysis by CAH1 involves an initial docking or binding with cell surface carbohydrates or phospholipid groups [39]. An haemolysin has been isolated from *Chiropsalmus quadrigatus* [40] and two hemolytic proteins having a sigmoidal dose-response curve activity were isolated from *Chironex fleckeri* [41,42]; this species has several haemolysins with varied molecular masses [31].

### 2.4. Hemolytic Scyphozoan Venoms

Among Scyphozoans, the cytolytic action of venom of the common marine Mediterranean jellyfish *Rhizostoma pulmo* was analysed. After soaking of jellyfish oral arms in distilled water two fractions were obtained: the first, rich in nematocysts was discarded, while the second, free of organelles and considered the extranematocystic portion, was tested on human RBC observing a low hemolytic activity [43] and subsequently analyzed by HPLC. Five peaks (a–e) were isolated by C18 preparative column, and the high molecular weight fractions were eluted by countercurrent technique with a flow rate of 5 mL/min. The cytolytic activity was evaluated on human RBC by both turbidity decrease test (at 700 nm) and haemoglobin release (at 418 nm) on 0.05% erythrocyte suspensions in 0.02 M tris-HCl buffer, containing 10 mM CaCl_2_ at pH 7.4. A concentration of 32 μg/mL of toxin induced complete hemolysis of erythrocytes in 10 min, thus suggesting a good capacity for binding to membranes [44]. 

The hemolytic activity of the giant jellyfish *Nemopilema nomurai* (Rhizostomeae) was assessed on cat, dog, human, rabbit and rat erythrocytes and showed a concentration-dependent activity starting from 10 μg/mL of protein equivalents; dog erythrocytes were the most sensitive (EC_50_ = 151 μg/mL) [45].

Purified cnidocyst extracts from fishing and mesenteric tentacles of *Cyanea capillata* (*Semaeostomeae*) induced hemolysis on human RBC with a difference between extracts coming from smaller or larger specimens. A complete hemolysis was caused by extracts (20 μg/well) from fishing tentacles of *C. capillata* with an umbrella diameter larger than 20 cm [46]. Furthermore, the erythrocyte lysis (HE_50_) induced by crude venom from mesenteric tentacles of large jellyfish was greater (98 μg/mL) than that induced from small medusae extracts (177 μg/mL). Therefore, the size of fishing tentacles and of oral arms, and nematocyst (A-isorhizas and O-isorhizas) number and size that correspond to the size of umbrella, were correlated with the cytolytic potency in differently-sized *Cyanea capillata* (L.) showing that the greater the specimen, the higher the produced hemolysis [47]. A concentration-dependent increase of hemolysis induced by extracts from *Cyanea capillata* tentacles was observed also in rat erythrocytes in the presence of Ca^2+^; this increase was attenuated by Ca^2+^ channel blockers such as Diltiazem, Verapamil and Nifedipine [48]. The hemolytic activity of extracts from *Cyanea lamarckii* was documented recently; the nematocystic extracts from mesenteric tentacles caused strong hemolytic effects, while extracts from fishing tentacles were less active [46]. 

The crude venom from *Pelagia noctiluca* was shown to induce hemolysis of chicken and rabbit but was not effective on fish red blood cells [49]. The hemolytic properties of *P. noctiluca* venom could be due to a pore-forming mechanism [50] and can be counteracted by osmotic protectants, such as carbohydrates, cations, proteases and antioxidants [51].

The proteic fractions responsible for the hemolytic properties of the nematocystic crude venom from *Pelagia noctiluca* were recently underlined [52] using teleost (*Carassius auratus*, freshwater; *Liza aurata*, marine) RBC. The nematocyst venom was used at various concentrations to evaluate the hemolytic activity and the stability of lysosomal membrane. Sphyngomyelin was shown to strongly inhibit the hemolytic activity. SDS-PAGE electrophoresis and high performance liquid chromatography (HPLC) showed that at least four protein fractions represent the active hemolytic components of crude venom. The crude venom from *Pelagia noctiluca* induced also lysosomal membrane destabilisation of fish RBC in both species; on the whole, *Carassius auratus* was more susceptible to jellyfish venom than *Liza aurata*. Contrary to what was reported in other articles, the authors state that crude venom does not cause oxidative stress because significant differences in glutathione (GSH) levels were not observed between control and treated cells, but it recognizes specific targets, such as sphyngomyelin, in RBC plasmatic membrane [52].

## 3. Cytotoxicity of Cnidarian Extracts on Cultured Cells

### 3.1. Cytotoxicity of Extracts from Octocorallia (Anthozoa)

Extracts from soft corals and gorgonians (*Anthozoa*: *Octocorallia*) have been widely studied and several of them were found to affect growth and survival of cultured cells. 

A number of papers concerned the study of extracts from *Clavularia* spp. (*Alcyionacea*). In the early 1980s, clavulones derived from the Japanese stolonifer *Clavularia viridis* were identified [53] and studied later for their activity on the growth of human cancer (HL-60 and HeLa) and normal (liver and lung fibroblasts) cultured cells found to be highly effective for antiproliferative and cytotoxic activity against HL-60 (IC_50_ = 0.4 μM or 0.2 μg/mL) and HeLa cells (significant cytotoxicity over 1.0 μM or 0.5 μg/mL); furthermore, clavulone was able to block the cells in G1-phase and to affect cell growth of HL-60 cells by inhibiting S-phase DNA synthesis [54]. Halogenated prostanoids (chlorovulone, bromovulone, and iodovulone) from *Clavularia viridis* were tested for antiproliferative and cytotoxic activities in cultured leukemic HL-60 cells; chlorovulone (IC_50_ growth inhibition = 0.03 μM or 0.01 μg/mL; cytotoxic effects >0.3 μM or 0.1 μg/mL) was highly effective being its activity stronger than that of prostaglandin A2; bromovulone and iodovulone showed comparable cytotoxic properties. The authors stated that “chlorovulone I transiently arrested the cell cycle progression from G1 to S after 24-h exposure to nontoxic concentrations (0.03 and 0.09 μM) and caused the lasting blockade of leukemia cells in G1 at the cytotoxic concentration” [55]. 

Marine diterpenoids (stolonidol and stolonidol monoacetate) isolated from *Clavularia* sp. showed strong cytotoxicity against P388 leukemia cells (both IC_50_s = 0.015 μg/mL); claenone, isolated from the same coral, was found to inhibit fertilized sea urchin (*Pseudocentrotus depressus*) eggs at a concentration of 2 μg/mL [56]. The induction of choline acetyl transferase (ChAT) by stolonidiol from *Clavularia* sp. was observed in cultured rat basal forebrain cells and in mouse clonal septal SN49 cells, a hybridoma cell line derived from primary cultured mouse basal forebrain cells [57]. This ChAT inducible activity on both primary cultures of cholinergic neurons and hybridoma suggested that stolonidol could act as a neurotrophic factor-like agent on the cholinergic nervous system.

Subsequently, Watanabe *et al.* [58] isolated five new halogenated prostanoids from *Clavularia viridis*; one of them was cytotoxic for human T lymphocyte leukemia cells (MOLT-4), human colorectal adenocarcinoma cancer cells (DLD-1), and human lung fibroblast (IMR-90) with IC_50_s of 0.52, 0.6, and 4.5 μg/mL, respectively.

A diterpene compound obtained through chromatography from the Formosan soft coral *Clavularia inflata* was found to be strongly cytotoxic to A-549, HT-29, and P-388 cell lines (ED_50_ values = 0.57, 0.31, and 0.052 μg/mL, respectively). Other five compounds were moderately cytotoxic to P-388 cells and little or nothing cytotoxic to A-549 and HT-29 cells [59].

The diterpene Sinugibberol extracted from *Silularia gibberosa* induced significant cytotoxicity to HT29 cells (ED_50_ = 0.5 μg/mL) and less damage to P388 cells (ED_50_ = 11.7 μg/mL) [60]. Iwashima *et al.* [61] isolated seven new diterpenoids having a cembrane skeleton from the Okinawan soft coral *Clavularia koellikeri*; One of these compounds (Compound 1) resulted cytotoxic to human colorectal adenocarcinoma cells (DLD-1), with an IC_50_ value of 4.2 μg/mL and a complete growth inhibition at 0.6 μM/mL, and affected the growth of MOLT-4 human T lymphocytic leukemia cells (IC_50_ = 0.9 μg/mL); similar results on the same cell lines were reported also for the diterpenoids kericembrenolides D and F. 

Mild cytotoxic activity was pointed out for a briariane diterpene lactone isolated from the New Guinean gorgonian *Solenopodium excavatum* that induced mild cytotoxicity (ED_50_ = 23 μg/mL) to P388 cells [62]. The diterpenoids echinoclerodane A and echinolabdane A were isolated from the Formosan gorgonian coral *Echinomuricea* sp. (Alcyonacea); echinoclerodane A was shown to exhibit moderate cytotoxicity against cultured MOLT-4 (human acute lymphoblastic leukemia), HL-60 (human acute promyelocytic leukemia), DLD-1 (human colorectal adenocarcinoma) and LoVo (human colorectal adenocarcinoma) cells, while K562 (human erythromyeloblastoid leukemia) and DU-145 (human prostate carcinoma) cells showed higher IC_50_ values. A moderate inhibition (35.4%) of elastase release by human neutrophils was observed at 10 μg/mL of Echinoclerodane A [63]. Echinolabdane A, a labdane-type diterpenoid derived from *Echinomuricea* sp. collected off the coast of southern Taiwan, was weakly cytotoxic *in vitro* for human acute promyelocytic leukemia cells HL-60 (IC_50_ = 19.1 μg/mL), and mildly inhibited superoxide anions generation by human neutrophils (inhibiting concentration IC_50_ > 10 μg/mL; percentage of inhibition at 10 μg/mL = 2.52 ± 3.02) as well as elastase release (IC_50_ =>10.0; percentage of inhibition = 1.83 ± 3.46) [64]. On the contrary, the sterol 6-epi-yonarasterol B isolated from the same coral significantly inhibited the generation of superoxide anions by human neutrophils (IC_50_ = 2.98 ± 0.29 μg/mL; percentage of inhibition = 89.76 ± 5.63) and the release of elastase (IC_50_ = 1.13 ± 0.55 μg/mL; percentage of inhibition = 95.54 ± 6.17 [64]. 

Steroids extracted from the gorgonian *Plexaurella grisea* were tested on tumour cell lines P 388 (mouse lymphoid neoplasm), A 549 (human lung carcinoma) and HT 29 (human colon carcinoma); some compounds showed a selective activity against HT 29 cells (ED_50_ = 0.1 μg/mL) [65]. The same research group reported that the organic extract of *Plexaurella grisea* specimens from Punta Cana (Dominican Republic) exhibited cytotoxicity against mouse lymphoma P-388, human lung carcinoma A-549, and human colon carcinoma HT-29 (IC_50_ = 2.5 μg/mL) cells, and described new compounds: five acyclic sesquiterpenes, namely (3*E*,5*E*)-3,7,11-trimethyl-9-oxododeca-1,3,5-triene (compound 3), (3*Z*,5*E*)-3,7,11-trimethyl-9-oxododeca-1,3,5-triene (compound 4), (3*E*)-6-acetoxy-3,11-dimethyl-7-methylidendodeca-1,3,10-triene (compound 5), (3*E*,5*E*)-7-hydroxy-3,7,11-trimethyldodeca-1,3,5,10-tetraene (compound 6), and (3*E*,5*E*,9*E*)-8,11-diacetoxy-3,7,11-trimethyldodeca-1,3,5,9-tetraene (compound 7), and two linear norsesquiterpenes, namely (2*E*,4*E*,7*Z*)-2,6,10-trimethylundeca-2,4,7,9-tetraenal (compound 8) and (2*E*,4*E*)-2,6,10-trimethylundeca-2,4,9-trienal (compound 9). Compounds 3, 4, 5, 8, and 9, were tested for cytotoxicity against P-388 mouse lymphoma cells, A-549 human lung carcinoma cells, HT-29 human colon carcinoma cells, and MEL-28 human melanoma cells. Compound 9 showed the greatest cytotoxic potential and was selective for P-388 cells (IC_50_ = 0.5 μg/mL), compound 8 resulted inactive (IC50 > 10 μg/mL), while other compunds induced mild cytotoxicity with IC_50_ values ranging from 2.5 to 5 μg/mL [66].

As concerns the cytotoxicity of Alcyoniidae derivatives, studies carried out in the late 1990s report that singardin, a heptacyclic norcembranoid dimer and the sesquiterpene guaianediol from *Sinularia gardineri*, showed cytotoxicity to murine leukemia P-388 (1 μg/mL), human lung carcinoma A-549 (2.5 μg/mL), human colon carcinoma HT-29 (5 μg/mL), and human melanoma MEL-28 (5 μg/mL) cells. Singardin was found also to have weak antifungal activity against *Candida albicans* and *Cryptococcus neoformans* [67]. Furthermore, an acylated spermidine isolated from the Pacific soft coral *Sinularia* sp. was found to be cytotoxic to P-388 cells (ED_50_ = 0.04 μg/mL) [68]. It is noteworthy that as early as the late 1970s, aqueous alcohol extracts and cembranolides extracted from *Sinularia flexibilis* were observed to have antineoplastic activity against P-388 lymphocytic leukemia [69]. Studies on cembranoid diterpenes sinuflexolide, dihydrosinuflexolide, and sinuflexibilin from *Sinularia flexibilis* were carried out by Duh *et al.* [70]; sinuflexolide and sinuflexibilin were significantly cytotoxic for A549, HT-29, KB, and P-388 cells (ED_50_ ranging from 0.16 to 1.73 μg/mL), while dihydrosinuflexolide affected significantly the growth of P-388 cells (ED_50_ = 3.86 μg/mL). Sphingolipids extracted from *Sinularia leptoclados* were found to have not cytotoxic properties *in vitro* against Vero cells at a concentration of 2 mg/mL and africanene from the same cnidarian exhibited *in vitro* cytotoxicity against DLAT (Dalton’s lymphoma ascites tumour) and EAC (Ehrlich ascites carcinoma) cells killing all treated cells at concentrations of >10 and >20 μg/mL, respectively [71]. Hexane extracts from *Sinularia inelegans* showed significant cytotoxicity against human lung adenocarcinoma (A549) and mouse lymphocytic leukemia (P-388) cells; a lobane diterpene (ineleganene) isolated from these extracts exhibited cytotoxicity against A549 cells (GI_50_ = 3.63 μg/mL) and P-388 cells (GI_50_ = 0.20 μg/mL) [72]. The cembranolide Capillolide from the soft coral *Sinularia capillosa* showed moderate cytotoxic activity against P-388 and L1210 cells with ED_50_ values of 15.0 and 18.5 μg/mL, respectively; other cembranolides gave ED_50_ values ranging from 1.5–10.0 μg/mL [73]. Recently, the cytotoxicity of cembranoids extracted from *Sinularia discrepans* was determined to human T-cell acute lymphoblastic leukemia (CCRF-CEM) and human colon adenocarcinoma (DLD-1) cells but neither cytotoxic activity nor growth inhibition (all IC_50_ > 20 μg/mL) were shown by these compounds. In addition, the anti-inflammatory activity *in vitro* of metabolites of such cembranoids was assessed using a macrophage cell line (RAW264.7). The inhibition of lipopolysaccharide (LPS)-induced up-regulation of inducible nitric oxide synthetase (iNOS) and cyclooxygenase-2 (COX-2) pro-inflammatory proteins in macrophages was examined and reduced levels of iNOS in comparison with controls (stimulated with LPS alone) as well as reducton of COX-2 expression induced by one of metabolites (named compound 5) were observed at the concentration of 10 μM. Thus, the possibility to utilize these compounds as anti-inflammatory agents was suggested [74].

Strong cytotoxicity on cells established from DBA/MC fibrosarcoma was exhibited by lemnalone, a ketone derivative of the sesquiterpenoid lemnalol isolated from the soft coral *Lemnalia tenuis* [75] and the antiinflammatory properties of Lemnalol (8-isopropyl-5-methyl-4-methylene-decahydro-1,5-cyclo-naphthalen-3-ol) isolated from *Lemnalia cervicorni* were investigated in lipopolysaccharide (LPS)-stimulated RAW 264.7 cells (murine macrophages). This natural compound was seen to inhibit significantly the expression of pro-inflammatory proteins, inducible nitric oxide synthase (iNOS) and cyclooxygenase-2 (COX-2) [76]. 

Cembrenolide diterpenes (sarcocrassolide, crassolide, 13-acetoxysarcocrassolide, denticulatolide), isolated by Duh *et al.* [77] from the soft coral *Sarcophyton crassocaule* Moser (*Alcyoniidae*) exhibited strong cytotoxicity against P-388 (mouse lymphocytic leukemia) cells showing ED_50_ values ranging from 0.14 to 0.38 μg/mL. The same effect was recorded for the steroid (24*S*)-24-methylcholestane-3β,5α,6β-triol whose ED_50_ accounted to 0.14 μg/mL. The growth inhibition induced by these compounds on other cell lines, A549 (human lung adenocarcinoma), HT-29 (human colon adenocarcinoma), and KB (human epidermoid carcinoma) was less effective, with ED_50_s ranging from 4.29 to 9.15 μg/mL. Another steroid (24ξ-methylcholestane-3β,5α,6β,25-tetraol-25-monoacetate) furnished higher ED_50_ values and was effective only on P-388 (3.96 μg/mL) and HT-29 (4.32 μg/mL) cells [77]. In a contemporary study, 10 different compounds extracted from the soft coral *Sarcophyton trocheliophorum* were tested for cytotoxicity on a panel of three cell lines, HL60 (human leukemia), M14 (skin melanoma), MCF7 (breast carcinoma), and on normal human peripheral blood lymphocytes; only polyhydroxysterol, 23,24-dimethylcholest-16(17)-*E*-en-3β,5α,6β,20(*S*)-tetraol caused strong cytotoxicity on cell lines with EC_50_ values ranging from 2.8 (HL60) to 4.9 (MCF7) μg/mL and exhibited weak toxicity to human lymphocytes; other compounds showed higher EC_50_s ranging from 10.4 to >100 μg/mL [78].

The cytotoxicity of six metabolites extracted from the Taiwanese Gorgonian coral *Subergorgia suberosa* was tested to KB (human nasalpharyngeal carcinoma) and HeLa (cervix carcinoma) cancer cells; subergorgic acid methyl ester moderately inhibited the growth of HeLa cells (ED_50_ = 4.3 μg/mL) while the other metabolites resulted not cytotoxic (ED_50_ > 10 μg/mL) [79]. Wang *et al.* [80] from the same species isolated four β-caryophyllene-derived sesquiterpenes alcohols (suberosols A, B, C and D), and two β-caryophyllene-derived sesqueterpene ketones (buddledins C and D); all metabolites resulted cytotoxic to P-388 cells (mouse lymphocytic leukemia) with ED_50_ values ranging from 2.1 to 7.4 μg/mL. The other two cell lines, A549 (human lung adenocarcinoma) and HT-29 (human colon adenocarcinoma), were sensitive to suberosols C and D and to both buddlesins (ED_50_ ranges: 3.8–8.9 μg/mL and 2.3–6.6 μg/mL, respectively) but were not affected by treatment with suberosols A and B (ED_50_ values > 50 μg/mL). 

Utilizing the same cell lines (P-388, A549, and HT-29), Wu *et al.* [81] studied the cytotoxicity of four polyoxygenated briarane-type diterpenoids (briaexcavatolides O, P, Q, R), isolated from the Taiwanese gorgonian *Briareum excavatum* (*Gorgonaceae*); one of these diterpenoids, Briaexcavatolide P, was found to be cytotoxic mainly to P-388 and HT-29 cancer cells (ED_50_s = 0.9 and 3.1 μg/mL, respectively) and was less active against A549 cells (ED_50_ = 4.8 μg/mL). The genus *Briareum* has been studied for cytotoxicity for decades. In fact, during the 1990s, crude extracts from *Briareum asbestinum*, a common inhabitant of shallow Caribbean reefs, were shown to be highly toxic to CHO-K1 cells at concentrations <25 μg/mL. Furthermore, four derivatives were significantly cytotoxic to the same cells with ED_50_ values of 3.35, 2.50, 3.55, and 4.82 μg/mL, respectively as well as active against *Klebsiella pneumoniae* [82]. Diterpenes 2β-acetoxy-2-(debutyryloxy)stecholide E, 9-deacetylstylatulide lactone, 4β-acetoxy-9-deacetylstylatulide lactone, brianthein W and 9-deacetylbriareolide H were isolated from the gorgonian *Briareum* sp.; their derivatives were found to be cytotoxic to P-388, KB, A-549, and HT-29 cancer cell lines [83]. Excavatolides A-E and brianolide from *Briareum excavatum* were studied for cytotoxicity. Three of them were found to be cytotoxic in particular to P-388 (ED_50_ = 0.3–1.8 μg/mL) but also to HT-29 (ED_50_ = 1.3–1.9 μg/mL) cancer cells and less effects were recorded against KB and A-549 cells [84]. Sung *et al.* [85] isolated eight new briarane-type diterpenes (excavatolides F-M) from the gorgonian *Briareum excavatum*. Only excavatolide M, with ED_50_ values ranging from 0.001 μg/mL (P-388) to 2.2 μg/mL (for HT-29) and excavatolide K, with ED_50_ values ranging from 0.9 μg/mL (for P-388) to 3.3 μg/mL (for KB), were cytotoxic to P-388, KB, A-549 and HT-29 cultured cells. Subsequently, Sung *et al.* [86] studied four new briarane diterpenes, briaexcavatolides K-N, and a known diterpene, compound 5, isolated from the Taiwanese gorgonian *Briareum excavatum*. Diterpenes K, M, and N resulted unactive for cytotoxicity against P-388 (mouse lymphocytic leukemia), A549 (human lung adenocarcinoma), and HT-29 (human colon adenocarcinoma) tumor cells; otherwise, Briaexcavatolide L was significantly cytotoxic to P-388 cells (ED_50_ = 0.5 μg/mL), and the compound 5 to both P-388 and HT-29 cells (ED_50_ = 0.4 and 1.1 μg/mL, respectively).

On the basis of the known lethality of crude extracts on brine shrimp (LC_50_ = 127 ppm) and cytotoxicity against P-388 cells (LC_50_ = 37 μg/mL), Rho *et al.* [87] isolated four new diterpenoids of the xenicane class (Acalycixeniolides C-F) from the gorgonian *Acalycigorgia inermis*. All compounds were cytotoxic to cultured human leukemia cells K562; Acalycixeniolide F showed the greatest cytotoxicity (LC_50_ = 0.2 μg/mL). The compounds C and E showed LC_50_ values of 1.6 and 4.7 μg/mL, respectively, while a weak cytotoxicity was shown by Acalycixeniolide D (LC_50_ = 52.0 μg/mL). The authors stated that Acalycixeniolide F “having a terminal dimethylvinyl moiety, exhibits cytotoxicity of an order of magnitude more potent than other xenicanes having an allene group at this position” and Acalycixeniolide D “bearing an α,β-unsaturated lactone group is considerably less active than the others” [87]. Subsequently, the same research group isolated eight diterpenes and norditerpenes, including five new xenicane metabolites (acalycixeniolides H-L), from the same gorgonian. 9-deoxyxeniolide A as well as the five new isolated compounds exhibited significant cytotoxicity to K562 human leukemia with LC_50_ values of 0.04, 3.9, 1.2, 2.0, 1.8, and 1.5 μg/mL, respectively; Acalycixeniolide E showed also antiangiogenic activity [88].

The soft coral *Alcyonium patagonicum* yielded a sterol (24-methylenecholest-4-ene-3β,6β-diol) that resulted cytotoxic against the P-388 cell line (IC_50_ = 1 μg/mL) [89]. Cytotoxic compounds were found also in subantarctic soft corals *Alcyonium paessleri*, a deep-living species collected near the South Georgia Islands. From this coral, 15 illudalane sesquiterpenoids, alcyopterosins A-O, were first isolated by Palermo *et al.* [90]; four of them were found to be mildly cytotoxic toward human tumor cell lines. Alcyopterosin A, C and H were effective against human colon carcinoma HT-29 cells with IC_50_ values of 10 μg/mL, and Alcyopterosin E affected human larynx carcinoma Hep-2 cells (IC_50_ = 13.5 μM) [90]. Another study reported that two sesquiterpenoids, paesslerins A and B, from the soft coral *Alcyonium paessleri,* showed moderate cytotoxicity against human tumor cell lines [91].

As concerns the genus *Eunicea*, four diterpenes (Edunone, Eduenone, Edudione and Edunol) isolated from the Caribbean gorgonian *Eunicea laciniata* showed weak cytotoxicity against HeLa cells with IC_50_ of 25, 50, 100 and 25 μg/mL, respectively [92] and diterpenoid cembranolides isolated from *Eunicea mammosa* were found to have moderate cytotoxicity against HeLa cells with IC_50_ values of 2.5 μg/mL for Uprolide D acetate, 5.0 μg/mL for Uprolide D, 3.0 μg/mL for Uprolide E acetate, and 5.1 μg/mL for Uprolide F diacetate. Uprolide D-acetate was cytotoxic also for human T-cell leukemia CCRF-CEM cells (IC_50_ = 7.0 μg/mL), HCT 116 colon cancer cells (IC_50_ = 7.0 μg/mL), and MCF-7 breast adenocarcinoma cells (IC_50_ = 0.6 μg/mL) [93]. The cembranoid diterpene asperdiol acetate from the Caribbean sea whip *Eunicea succinea* showed cytotoxic properties resulting in GI_50_ values of 6.25 × 10^−7^ M against SNB-75 CNS cancer cells and 8.28 × 10^−6^ against M14 (melanoma) and HS 578T (breast cancer) cell lines [94]. Eight γ-cembranolide-type diterpenes and a new saponin were isolated from the gorgonian octocoral *Eunicea pinta* by Shi *et al.* [95]. The diterpene 12-Epieupalmerone was cytotoxic to non-small cell lung cancer cells NCI-H332M (IC_50_ = 0.90 μg/mL) and also to renal cancer cells TK-10 (IC_50_ = 0.13 μg/mL) and Uprolide H strongly inhibited the growth of human T lymphocitic leukemia cells MOLT-4 (IC_50_ = 0.01 μg/mL) and SR (IC_50_ = 0.07 μg/mL). The saponin was cytotoxic only to renal cancer cells A498 (IC_50_ = 4.2 μg/mL), ACHN (IC_50_ = 2.8 μg/mL) and CAKI-1 (IC_50_ = 6.6 μg/mL) [95].

Nephtheoxydiol from the soft coral *Nephthea* sp. exhibited a significant growth-inhibitory effect on B-16 melanoma cells with IC_50_ of 0.1 μg/mL [96]. In a subsequent study, six sterols isolated from the same coral were studied for cytotoxicity. Five of them exhibited significant cytotoxicity and affected the growth of A549 (human lung adenocarcinoma), HT-29 (human colon adenocarcinoma), KB (human epidermoid carcinoma), and P-388 (murine lymphocytic leukemia) cell lines (ED_50_ values ranging from 0.07 to 1.76 μg/mL); the sixth compound was significantly cytotoxic only to P-388 and HT-29 cells [97].

On the basis of the known occurrence of bioactive terpenoids in soft corals of the genus *Xenia* (Alcyonacea), several diterpenoids were isolated and described in two different papers [98,99]. In the first paper, eight new compounds named blumiolide-A (1), blumiolide-B (2), 9-deoxy-isoxeniolide-A (3), 9-deoxy-7,8-epoxy-isoxeniolide-A (4), 9-deacetoxy-7,8-epoxy-13-epi-xenicin (5), 9-deoxy-7,8-epoxy-xeniolide-A (6), blumiolide-C (7), and blumicin-A (8), were isolated from *Xenia blumi* and were found to be cytotoxic to A549 (human lung adenocarcinoma), HT-29 (human colon adenocarcinoma), and P-388 (mouse lymphocytic leukemia) cells with ED_50_ values ranging from 0.2 (compound 7) to 6.9 (compound 6) μg/mL for P-388 and from 0.5 (compound 7) to 8.7 (compound 3) μg/mL for HT-29. Compounds 5 and 8 were practically inactive on both cell lines and compound 3 showed ED_50_ values >20 μg/mL on P-388 cells [98]. In the second paper, 11 diterpenoids, umbellacins A-G (1–7), 14,15-epoxy-xeniolide H (8), 3-acetyl-14,15-epoxy-xeniolide H (9), and umbellacins H and I (10, 11) were isolated from the soft coral *Xenia umbellata* (*Alcyonacea*). Compounds 2, 4, 5, 6, 10 and 11 were cytotoxic *in vitro* against murine P-388 lymphocytic leukemia cells with ED_50_ values of 1.6, 4.2, 3.8, 3.7, 3.4, and 3.6 μg/mL, respectively, but they were not cytotoxic to human lung adenocarcinoma cells (A549) and human colon adenocarcinoma cells (HT-29) [99].

Punaglandins—highly functionalized prostanoids provided with anti-inflammatory and antitumor activity—were seen to be active against cultured L1210 mouse leukemia cells [100]; 19 of these compounds produced by the Hawaiian octocoral *Telesto riisei* (*Telestacea*) were described by Baker and Scheuer [101]. The activity of punaglandins both *in vitro* and *in vivo* against Ehrlich ascites cells was stronger than that of prostaglandins and the induced cytotoxicity almost equalised that from vincristine; modified compounds were shown to enhance the mineralization of human osteoblasts *in vitro* [102]. Other compounds isolated from the octocoral *Telesto riisei N*-(2-phenylethyl)-9-oxohexadecacarboxamide and *N*-(2-phenylethyl)-9-hydroxyhexadecacarboxamide, acyl derivatives of β-phenylethylamine, and two tetrahydroxysterols were found to be mildly toxic to murine leukemia cells (P-388) with ED_50_ ranging from 1.3 to 2.4 μg/mL [103].

A screening with crude extracts from octocorals *Carijoa* sp. and *Lophogorgia* sp. and with other unidentified gorgonians showed that a high percentage (30%) displayed cytotoxic activities to cultured MCF-7 (breast), B16 (melanoma) and HCT8 (colon) cancer cells with values of growth inhibition in some cases (mainly for *Carijoa* sp.) higher than 75%. The antimycotic activity against *Candida albicans* was observed for one unidentified gorgonian [104].

A modified steroid (18-acetoxipregna-1,4,20-trien-3-one) isolated from *Carijoa riisei* was shown to be cytotoxic (IC_50_ = 10.6 μg/mL) to mammalian macrophages [30]. Another study [105] showed that this steroid was moderately cytotoxic against cancer cells with IC_50_ values of 12.4 μg/mL when tested on leukemia (HL60) cells, 14.4 μg/mL for SF295 glioblastoma cells, 22.0 μg/mL for colon HCT8 cells and 23.1 μg/mL for MDA.MB.435 ductal carcinoma cells, to date classified as melanoma. A pregnane steroid and two analogues isolated from *Carijoa* sp. exhibited cytotoxicity against Bel-7402 cells (human hepatoma) with IC_50_ values of 9.33, 11.02 and 18.68 µM, respectively [106].

As concerns the genus *Lobophytum*, Lobohedleolide, containing the α,β-unsaturated carboxylic acid system, isolated from the Japanese soft coral *Lobophytum hedleyi*, was shown to cause growth inhibition of HeLa cells *in vitro* [107]. Two cytotoxic cembranolides—lobomichaolide and crassolide—were studied in *Lobophytum michaelae* (*Alcyoniidae*); they exhibited significant cytotoxicity against A-549 human lung adenocarcinoma cells (ED_50_ = 0.38 and 0.39 μg/mL, respectively), HT-29 human colon adenocarcinoma cells (ED_50_ = 0.37 and 0.26 μg/mL, respectively), KB human nasopharyngeal carcinoma cells (ED_50_ = 0.59 and 0.85 μg/mL, respectively), and P-388 mouse lymphocytic leukemia cells (ED_50_ = 0.34 and 0.08 μg/mL, respectively) [108]. Later on, hexane extracts from the soft coral *Lobophytum crassum* showed significant cytotoxicity against human lung adenocarcinoma (A549), human colon adenocarcinoma (HT-29), human epidermoid carcinoma (KB), and mouse lymphocytic leukemia (P-388) cells. A cembrane diterpene (lobocrassolide) and a cembrenolide (lobohedleolide) were isolated from these extracts. Both compounds showed cytotoxicity; in particular, lobocrassolide was cytotoxic for all utilized cultured cells with ED_50_ values of 2.99, 2.70, 2.91, and 0.012 μg/mL for A549, HT-29, KB, and P-388 cells, respectively. Lobohedleolide was shown to be cytotoxic only to P-388 cells (ED_50_ = 2.44 μg/mL) [109].

Other octocorals and their extracts were studied for cytotoxicity from as early as the 1990s. 9,11-secosterol from the soft coral *Gersemia fruticosa* was found to be cytotoxic as well as to inhibit the growth of cultured human leukemia K562, human cervical cancer HeLa, and Ehrlich ascites tumor cells with IC_50_ values below 10 μM. The action mechanism seems to be linked to the induction of mitotic alterations with the blockade of cell cycle progression and accumulation of cells in the metaphase of mitosis. After treatment, Ehrlich tumor cells pursued DNA synthesis without entry into mitosis producing cells with high DNA ploidy [110]. Palmonines from the gorgonian *Eunicella verrucosa* were found to be weakly cytotoxic to murine (P-388 lymphoma), and human (A549 lung carcinoma, HT29 colon carcinoma, and MEL28 melanoma) cancer cells. Testings with these cell cultures furnished ED_50_ values of 10 μg/mL or higher in all cases; only the palmonine B was observed to be active against P-388 and MEL28 cells (ED_50_ = 5 μg/mL). On the basis of these results, the authors stated that “palmonines seem to offer one more example of the low potential pharmaceutical activities of compounds from octocorals” [111]. In 1998, from the gorgonian *Muricella* sp., five 9,10-secosteroids (astrogorgiadiol and calicoferols F-I) were studied for cytotoxicity by Seo *et al.* [112]. These compounds exhibited significant cytotoxicity against K-562 human leukemia cells with LC_50_ values of 12.1, 3.2, 2.1, 10.7, and 9.6 μg/mL, respectively [112]. The glycoside 19-norpregna-1,3,5(10),20-tetraen-3-*O*-α-fucopyranoside isolated from the soft coral *Scleronephthya pallida* was cytotoxic to the breast cancer cell line (BCA-1) with ED_50_ of 10 μg/mL; furthermore, this compound inhibited the growth of *Plasmodium falciparum* [113]. The sesquiterpene suberosenone and its dimer alertenone were extracted from the gorgonian *Alertigorgia* sp. The first compound caused strong growth inhibition of nonsmall cell lung (A549, HOP-92) and CNS (SF-295, SF-539, SNB-19) tumor cell lines, with IC_50_ ranging from 0.002 to 1.63 μg/mL, of melanoma cell lines (LOX, M14, MALME-3M) with IC_50_ ranging from 0.006 to 0.01 μg/mL, and of ovarian (OVCAR-3) and breast cell lines (MCF7), with IC_50_ of 0.02 and 0.43 μg/mL, respectively. Alertenone was practically devoid of cytotoxicity showing IC_50_ values ranging from 35 to >100 μg/mL [114]. Three steroids isolated from *Leptogorgia sarmentosa* showed significant but non selective cytotoxicity against suspension cultures of mouse lymphoid neoplasm (P-388) and monolayer cultures of human lung carcinoma (A 549), human colon carcinoma (HT 29), and human melanoma (MEL 28) with ED_50_ values of 1 μg/mL for all cases [115].

Six sesquiterpene metabolites (suberosenol A, suberosenol B, suberosanone, suberosenol A acetate, suberosenol B acetate and subergorgic acid) from the gorgonian *Isis hippuris* were studied by Sheu *et al.* [116]. Excluding subergorgic acid (ED_50_ values ranging from 13.3 to >50 μg/mL), all compounds showed significant cytotoxicity toward mouse lymphocytic leukemia (P-388), human lung adenocarcinoma (A549), and human colon adenocarcinoma (HT-29) cells with ED_50_s ranging from values lower than 5.0 × 10^−6^ μg/mL to 3.6 × 10^−1^ μg/mL except for suberosenol B that furnished ED_50_ results between 0.2 and 3.4 μg/mL. On the whole, suberosenol A was the most active compound showing high cytotoxicity toward all utilized cancer cells. The authors remark that this compound “contains a β-hydroxyl group at the allylic position of the 5,6-double bond” and suggest that “the molecular skeleton, not the functionalities, is the main factor for the potent cytotoxicity of these suberosane terpenoids” [116].

Three compounds, among which a new sesquiterpenoid (junceol A) and two known diterpenoids (sclerophytin A and cladiellisin), were isolated by Chen *et al.* [117] from sea pen octocoral *Virgularia juncea* and resulted cytotoxic to P-388 cancer cells showing ED_50_ values of 5.1, 2.3, and 2.0 μg/mL, respectively. A new briarane, juncenolide C, isolated from the Taiwanese red gorgonian *Junceella juncea* (*Alcyonacea*) exhibited mild cytotoxicity against human liver carcinoma HEPA 59T/VGH at a concentration of 6.6 μg/mL and oral epidermoid carcinoma cells (KB) at a concentration of 7.8 μg/mL [118]. (*Z*)-Sarcodictyin A from the soft coral *Bellonella albiflora* (*Alcyonacea*) collected from southern Japan was highly cytotoxic to human cervix HeLa tumour cells (IC_50_ = 90 ng/mL); other compounds (eleutherobin and (*Z*)-eleutherobin) from the same cnidarian showed IC_50_ values of 17 ng/mL [119]. Yoshikawa *et al.* [120] isolated two polyhydroxylated sterols, named dendronesterol A and B, from the octocoral *Dendronephthya gigantea*. Dendronesterol B was found to exhibit weak cytotoxicity toward lymphocytic leukemia cells (L1210) with IC_50_ value of 5.2 μg/mL. Guaiazulene-related pigments from gorgonians are known to have antifungal, antitumor, antibacterial and immunoregulatory activity as well as antiproliferative effects on fertilized sea urchin and ascidian eggs [121]. On this basis, three linderazulenes (compounds 1–3) isolated from the deep-sea gorgonian *Paramuricea* sp. (*Alcyonacea*) were tested for their cytotoxicity against P388 murine leukemia cells and PANC-1 pancreatic cells. The IC_50_ values calculated after treatment of P388 cells were 18.8, 2.7, and 15.6 μg/mL, respectively. Compound 2 was moderately cyotoxic (IC_50_ = 18.7 μg/mL) also to PANC-1 cells [121]. 

Chao *et al.* [122] isolated three steroidal carboxylic acids (paraminabic acids A–C) from the Formosan soft coral *Paraminabea acronocephala*. The compound C was highly cytotoxic (IC_50_ values ranging from 2.05 to 2.83 μg/mL) to cancer cell lines Hep3B, MDA-MB-231, MCF-7 and A-549.

**Table 1:** Cytotoxicity to different cell lines of compounds extracted from Octocorallia (Anthozoa).

Species	Compound or material	Cells	Tissue/organ/histology	Organism	IC_50_–ED_50_ (μg/mL)	Ref.
*Acalycigorgia inermis*	Xenicane diterpenoids	K562	Leukemia	Human	0.2–52.0	[87,123]
*Acalycigorgia inermis*	Xenicane diterpenoids	K562	Leukemia	Human	0.04–3.9	[88]
*Alcyonum patagonicum*	Dihydrohy sterol	P388	Lymphoma	Mouse	1.00	[89]
*Alcyonium paessleri*	Sesquiterpenoids	HT-29	Colon carcinoma	Human	10.0	[90]
		Hep-2	Larynx carcinoma	Human	13.5	
*Alertigorgia* sp.	Suberosenone (sesquiterpene)	A-549	Lung adenocarcinoma	Human	1.63	[114]
		HOP-92	Lung adenocarcinoma	Human	0.11	
		SF-295	Glioblastoma	Human	0.03	
		SF-539	Gliosarcoma	Human	0.002	
		SNB-19	Glioblastoma	Human	0.006	
		LOX	Melanoma	Human	0.006	
		M14	Melanoma	Human	0.010	
		MALME.3M	Melanoma	Human	0.008	
		OVCAR.3	Ovarian adenocarcinoma	Human	0.02	
		MCF7	Breast adenocarcinoma	Human	0.43	
*Bellonella albiflora*	Diterpenoids	HeLa	Cervix carcinoma	Human	17.0–90.0 (×)	[119]
*Briareum excavatum*	Briarane-type diterpenoid	P388	Lymphoma	Mouse	0.9	[81]
		A549	Lung adenocarcinoma	Human	4.8	
		HT-29	Colon adenocarcinoma	Human	3.1	
*Briareum excavatum*	Diterpenes	A549	Lung adenocarcinoma	Human	1.2–>50	[84]
		HT-29	Colon adenocarcinoma	Human	1.3–>50	
		KB	Epidermoid carcinoma (#)	Human	0.8–>50	
		P388	Lymphoma	Mouse	0.3–>50	
*Briareum excavatum*	Briarane diterpenes	A549	Lung adenocarcinoma	Human	0.1–>50	[85]
		HT-29	Colon adenocarcinoma	Human	1.3–>50	
		KB	Epidermoid carcinoma (#)	Human	1.0->50	
		P388	Lymphoma	Mouse	0.001– >50	
*Briareum excavatum*	Briarane diterpenes	P388	Lymphoma	Mouse	0.40– 0.50	[86]
		HT-29	Colon adenocarcinoma	Human	1.1	
*Briareum asbestinum*	Asbestinin diterpenes	CHO-K1	Ovary (normal)	Chinese hamster	2.50–4.82	[82]
*Briareum* sp.	Diterpenes	A549	Lung adenocarcinoma	Human	10.35–>50	[83]
		HT-29	Colon adenocarcinoma	Human	0.29–>50	
		KB	Epidermoid carcinoma (#)	Human	0.27–>50	
		P388	Lymphoma	Mouse	0.28–>50	
*Carijoa riisei*	Steroid	-	Macrophages	Mouse	10.6	[30]
*Carijoa riisei*	Steroid	SF295	Glioblastoma	Human	14.4	[105]
		MDA.MB.435	Ductal carcinoma (°)	Human	23.1	
		HCT8	Colon adenocarcinoma	Human	22.0	
		HL60	Promyelocytic leukemia	Human	12.4	
*Carijoa* (*Telesto*) *riisei*	Riiseins (steroidal glycosides)	HCT-116	Colon adenocarcinoma	Human	2.0	[124]
*Telesto riisei*	Amides	P388	Lymphoma	Mouse	2.1–2.2	[103]
	Sterols				1.3–2.4	
*Carijoa* sp.	Steroids	Bel-7402	Hepatoma	Human	9.33–18.68	[106]
*Clavularia inflata*	Dolabellane diterpene	A-549	Lung adenocarcinoma	Human	0.57	[59]
		HT-29	Colon adenocarcinoma	Human	0.31	
		P388	Lymphocytic leukemia	Mouse	0.052	
*Clavularia koellikeri*	Cembrane-type diterpenoid	DLD-1MOLT-4	Colorectal adenocarcinoma	Human	4.2	[61]
			T lymphocytic leukemia	Human	0.9	
*Clavularia viridis*	Clavulones	HL60	Promyelocytic leukemia	Human	0.2	[54]
		HeLa	Cervix adenocarcinoma	Human	0.5	
*Clavularia viridis*	Chlorovulone	HL60	Promyelocytic leukemia	Human	0.01	[55]
*Clavularia viridis*	Halogenated prostanoid (7-Acetoxy-7,8-dihydroiodovulone I)	MOLT-4	T lymphocytic leukemia	Human	0.52	[58]
		DLD-1	Colorectal adenocarcinoma	Human	0.6	
		IMR-90	Lung fibroblasts	Human	4.5	
*Clavularia* sp*.*	Stolonidol and Stolonidol monoacetate	P388	Lymphoma	Mouse	0.015	[56]
*Dendronephthya gigantea*	Dendronesterol B (sterol)	L1210	Lymphocytic leukemia	Mouse	5.2	[120]
*Echinomuricea* sp.	Diterpenoid	MOLT-4	Lymphoblastic leukemia	Human	13.18 (^)	[63]
		HL-60	Promyelocytic leukemia	Human	14.89 (^)	
		DLD-1	Colorectal adenocarcinoma	Human	23.44 (^)	
		LoVo	Colorectal adenocarcinoma	Human	21.69 (^)	
		K562	Erythromyeloblastoid leukemia	Human	37.05 (^)	
		DU-145	Prostate carcinoma	Human	53.93 (^)	
*Echinomuricea* sp.	Diterpenoid	HL-60	Promyelocytic leukemia	Human	19.1	[64]
*Eunicea laciniata*	Dolabellane diterpenes	HeLa	Cervix carcinoma	Human	25.0–100.0	[92]
*Eunicea mammosa*	Cembranolide diterpenoids	HeLa	Cervix carcinoma	Human	2.5–5.1	[93]
		CCRF-CEM	T-cell leukemia	Human	7.0	
		HCT 116	Colon cancer	Human	7.0	
		MCF-7	Breast adenocarcinoma	Human	0.6	
*Eunicea pinta*	γ-cembranolide-type diterpene (12-Epieupalmerone)	NCI-H322M	Non-small cell lung cancer	Human	0.90	[95]
		TK-10	Renal cancer	Human	0.13	
	Uprolide H (diterpene)	MOLT-4	T lymphocytic leukemia	Human	0.01	
		SR	Large cell immunoblastic lymphoma	Human	0.07	
	Saponin	A498	Renal cancer	Human	4.2	
		ACHN	Renal cancer	Human	2.8	
		CAKI-1	Renal cancer	Human	6.6	
*Eunicea succinea*	Asperdiol acetate (diterpene)	SNB-75	CNS cancer	Human	6.25 × 10^−7^ (* +)	[94]
		M14	Melanoma	Human	8.28 × 10^−6^ (* +)	
		HS 578T	Breast cancer	Human	8.28 × 10^−6^ (* +)	
*Eunicella verrucosa*	Palmonine B (diterpene)	MEL28	Melanoma	Human	5.0	[111]
		P388	Lymphoma	Mouse	5.0	
*Isis hippuris*	Sesquiterpenes	A549	Lung adenocarcinoma	Human	0.005–>50	[116]
		HT-29	Colon adenocarcinoma	Human	<0.000005–>50	
		P388	Lymphoma	Mouse	<0.000005–13.3	
*Lemnalia tenuis*	Lemnalone	DBA/MC	Fibrosarcoma	Mouse	2.5–40 (**)	[75]
*Leptogorgia sarmentosa*	Steroids	A549	Lung adenocarcinoma	Human	1	[115]
		HT-29	Colon adenocarcinoma	Human	1	
		MEL 28	Melanoma	Human	1	
		P388	Lymphoma	Mouse	1	
*Lobophytum crassum*	Diterpenes	A549	Lung adenocarcinoma	Human	0.012–2.99	[109]
		HT-29	Colon adenocarcinoma	Human		
		KB	Epidermoid carcinoma (#)	Human		
		P388	Lymphoma	Mouse		
*Lobophytum michaelae*	Cembranolides	A549	Lung adenocarcinoma	Human	0.38–0.39	[108]
		HT-29	Colon adenocarcinoma	Human	0.26–0.37	
		KB	Epidermoid carcinoma (#)	Human	0.59–0.85	
		P388	Lymphoma	Mouse	0.08–0.34	
*Muricella* sp.	Secosteroids	K-562	Leukemia	Human	2.1–12.1	[112]
*Nephthea brassica*	Brassicolene (diterpenoid)	A-549	Lung adenocarcinoma	Human	3.62	[125]
		P388	Lymphoma	Mouse	0.86	
*Nephthea erecta*	Sterols	A549	Lung adenocarcinoma	Human	0.41–4.09	[97]
		HT-29	Colon adenocarcinoma	Human	0.1–3.34	
		KB	Epidermoid carcinoma (#)	Human	0.38–>50	
		P388	Lymphoma	Mouse	0.07–0.45	
*Nephthea* sp.	Nephtheoxydiol (sesquiterpene)	B-16	Melanoma	Mouse	0.1	[96]
*Pachyclavularia violacea*	Pachyclavulariolide F (diterpenoid)	P388	Lymphoma	Mouse	1.0	[126]
*Paraminabea acronocephala*	Paraminabic acid C (steroidal carboxylic acid)	Hep G2	Liver hepatocellular carcinoma	Human	13.6–19.8	[122]
		Hep 3B	Liver hepatocellular carcinoma	Human	2.83–>20	
		MDA.MB.231	Breast adenocarcinoma	Human	2.25–>20	
		MCF-7	Breast adenocarcinoma	Human	2.23–>20	
		A-549	Lung adenocarcinoma	Human	2.05–>20	
*Paramuricea* sp.	Linderazulenes (terpenes)	P388	Lymphoma	Mouse	2.7–18.8	[121]
		PANC-1	Pancreatic carcinoma	Human	18.7	
*Pseudopterogorgia americana*	Secogorgosterols	LnCap	Prostate carcinoma	Human	15.5	[127]
		Calu-3	Lung adenocarcinoma	Human	11.0	
*Plexaurella grisea*	Polyhydroxylated sterols	P388	Lymphoma	Mouse	>1	[65]
		A549	Lung carcinoma	Human	1	
		HT29	Colon carcinoma	Human	0.1–1	
*Plexaurella grisea*	Linear norsesquiterpenes	P388	Lymphoma	Mouse	0.5–5	[66]
	Acylic sesquiterpenes	A549	Lung carcinoma	Human		
		HT29	Colon carcinoma	Human		
		MEL-28	Melanoma	Human		
*Sarcophyton crassocaule*	Cembrenolide diterpenes	A549	Lung adenocarcinoma	Human	4.29–8.31	[77]
		HT-29	Colon adenocarcinoma	Human	4.97–7.55	
		KB	Epidermoid carcinoma (#)	Human	6.29–9.15	
		P388	Lymphoma	Mouse	0.14–0.38	
	Steroids	A549	Lung adenocarcinoma	Human	6.26–22.43	
		HT-29	Colon adenocarcinoma	Human	4.32–8.35	
		KBP388	Epidermoid carcinoma (#)	Human	5.38–26.84	
			Lymphoma	Mouse	0.14–3.96	
*Sarcophyton trocheliophorum*	Polyhydroxysterol	HL60	Leukemia	Human	2.8	[78]
		M14	Melanoma	Human	4.3	
		MCF7	Breast carcinoma	Human	4.9	
*Scleronephthya pallida*	Pregnane steroids	BCA-1	Breast cancer	Human	10.0	[113]
*Sinularia capillosa*	Cembranolide (capillolide)	P388	Lymphoma	Mouse	15.0	[73]
		L1210	Lymphocytic leukemia	Mouse	18.5	
*Sinularia capillosa*	Cembranolides	P388	Lymphoma	Mouse	1.5–8.5	[73]
		L1210	Lymphocytic leukemia	Mouse	3.0–10.0	
*Sinularia flexibilis*	Cembranoid diterpenes	A549	Lung adenocarcinoma	Human	0.68–16.8	[70]
		HT-29	Colon adenocarcinoma	Human	0.22–32.4	
		KB	Epidermoid carcinoma (#)	Human	0.46–>50	
		P388	Lymphoma	Mouse	0.16–3.86	
*Sinularia gibberosa*	Diterpene (sinugibberol)	HT29	Colon adenocarcinoma	Human	0.50	[60]
		P388	lymphoma	Mouse	11.7	
*Sinularia inelegans*	Diterpene (ineleganene)	A549	Lung adenocarcinoma	Human	3.63 (*)	[72]
		P388	Lymphoma	Mouse	0.20 (*)	
*Sinularia* sp.	Acylated spermidine	P388	Lymphoma	Mouse	0.04	[68]
*Sinularia* sp.	Sterols	A549	Lung adenocarcinoma	Human	2.7–10.8	[128]
		HT-29	Colon adenocarcinoma	Human	0.7–1.5	
		KB	Epidermoid carcinoma (#)	Human	1.9–>50	
		P388	Lymphoma	Mouse	0.4–8.3	
*Subergorgia suberosa*	Sesquiterpene (Subergorgic acid methyl ester)	HeLa	Cervix carcinoma	Human	4.3	[79]
*Subergorgia suberosa*	Sesquiterpene alcohols	P388	Lymphoma	Mouse	2.1–7.4	[80]
		A549	Lung adenocarcinoma	Human	4.2–>50	
		HT-29	Colon adenocarcinoma	Human	2.3–>50	
	Sesquiterpene chetones	P388	Lymphoma	Mouse	4.6–6.3	
		A549	Lung adenocarcinoma	Human	3.8–8.9	
		HT-29	Colon adenocarcinoma	Human	3.6–6.6	
*Virgularia juncea*	Sesquiterpenoid	P388	Lymphoma	Mouse	5.1	[117]
	Diterpenoids	P388	Lymphoma	Mouse	2.0–2.3	
*Xenia blumi*	Diterpenoids	HT-29	Colon adenocarcinoma	Human	0.5–>20	[98]
		P388	Lymphoma	Mouse	0.2–>20	
*Xenia umbellata*	Diterpenoids	P388	Lymphoma	Mouse	1.6–3.8 (§)	[99]

(*) values expressed as GI_50_; (^) Values expressed as μM; (+) Values expressed as M; (×) Values expressed as ng/mL; (**) tested concentrations; IC/ED_50_ values not indicated; (§) only active compounds; (°) to date considered a melanoma; (#) to date considered a HeLa cell contaminant.

In a comprehensive review, Mayer and Gustafson [129] reviewed the available data of the antitumor and cytotoxic properties of 143 marine natural products among which some were derived from cnidarians; in particular, this paper reports that some diterpenes from corals were found to be active against human and murine tumour cell lines causing growth inhibition or cytotoxicity in the concentration range of 0.012–22.4 μg/mL [72,77,109,125,126]. Gorgonian and coral steroids were active in the concentration range of 0.4–18.43 μg/mL on human and murine tumor cells [115,123,127,128]; steroidal glycosides (riiseins A and B) from coral were found to be active to inhibit cell growth of human tumours at a concentration of 2.0 μg/mL [124] and a gorgonian sesquiterpene affected cell growth of murine and human tumour cell lines at concentrations of 5 × 10^−6^–50 μg/mL [128]. 

Table 1 shows a summary of data pertinent to the considered papers.

### 3.2. Cytotoxicity of Extracts from Hexacorallia (Anthozoa)

The studies about the cytotoxicity of hexacoral extracts using cultured cells are remarkably scarcer than those about octocorals. A Zoanthoxanthin alkaloid from zoanthid corals *Epizoanthus* sp. was shown to be cytotoxic *in vitro* against HCT8 human colon adenocarcinoma (IC_50_ = 1.61 μg/mL), A549 human lung carcinoma (IC_50_ = 2.38 μg/mL), HT29 human colon adenocarcinoma (IC_50_ = 0.824 μg/mL) and P-388 mouse lymphocytic leukemia (IC_50_ = 1.77 μg/mL) cells [130]. A fractionated extract of the stony coral *Tubastrea faulkneri* (Scleractinia) furnished macrolides and indole derivatives; among these compounds the macrolides mycalolides C and D induced modest cytotoxicity against 60 human tumor cell lines showing average LC_50_ values of 2.5 and 0.6 μM, respectively [131]. From eggs of the scleractinian coral *Montipora digitata*, the polyacetylene carboxylic acids montiporic acid A and B were isolated. These compounds exhibited cytotoxicity against P-388 murine leukemia cells with IC_50_ value of 12.0 μg/mL, and were active against bacteria *Escherichia coli* (IC_50_ = 5.0 μg/mL) [132]. Six acetylenic compounds have been isolated from the stony coral *Montipora* sp.; two of them (Compounds 1 and 3) showed high cytotoxicity (ranges of 1.4–3.7 μg/mL and 1.5–5.2 μg/mL, respectively) against SKOV-3 (human ovarian cancer), SK-MEL-2 (human skin cancer), XF498 (human CNS cancer), and HCT15 (human colon cancer) cell lines but had no activity on A549 (human lung cancer) cells [133]. 

Ten new and four known diacetylenes were isolated by a Korean Research Group from a fraction of the methanolic extract from the stony coral *Montipora* sp. active on brine shrimp [134]; a 15th compound, Montiporyne A, induced apoptosis in human HCT116 colon tumor cells with an increase of 19% of the apoptotic fraction in cells treated with 100 μg/mL for 24 h. These compounds were tested for cytotoxicity against five human cancer cell lines (A549: human lung cancer; SK-OV-3: human ovarian cancer; SK-MEL-2: human skin cancer; XF498: human CNS cancer; HCT15: human colon cancer); Montiporyne I resulted the most cytotoxic compound with ED_50_ values ranging from 1.40 to 4.17 μg/mL and showed significant cytotoxicity particularly against human ovarian cancer (SK-OV-3) and human skin cancer (SK-MEL-2) cells. The Montiporynes J, K and L as well as a diacetylene (compound 8) were also highly cytotoxic. The authors stated that “diacetylenes with the β-hydroxy ketone functionality were found to be more active” [134]. 

Palytoxin (PTX), at the beginning isolated in the hexacoral *Palythoa toxica* [8], is one of the most poisonous biotoxins [135,136] and has several important features: it is a human and mouse skin irritant, it was indicated to be a tumor promoter and to exert its activity extracellularly [137], and it is able to induce neurotoxicity, rhabdomyolysis and cardiovascular collapse *in vivo* on several mammals, including humans [138], as well as to modify the normal function of different biological systems [135]. PTX is a very complex molecule that presents both lipophilic and hydrophilic areas [139]. It was reported to affect cation transport across the plasma membranes [140,141,142] and to interact with Na^+^,K^+^-ATPase converting this ion pump into a non-specific cation channel and consequently causing perturbation of Na^+^, K^+^, Ca^2+^ and H^+^ ion fluxes resulting in cytotoxicity, cytolysis and cell death [135].

The effects of PTX were studied on human bronchial epithelial cells showing that it does not induce squamous differentiation of normal cells; the cytotoxicity of PTX does not change when normal human bronchial epithelial cells, human lung tumors, and human bronchial epithelial cells immortalized by infection with adenovirus 12-SV40 are used. Furthermore, PTX does not induce a change in free cytosolic Ca^2+^ concentration of BEAS-2B cells [143]. PTX was shown to induce ion currents (channels permeable to Na^+^ and K^+^ and slightly permeable to Ca^2+^, choline and tetramethylammonium) in mouse neuroblastoma cells [144]. As a matter of fact, the activity on cation transport is commonly recognized to be PTX’s main toxic mechanism [145].

PTX stimulates weak production of superoxide radicals by human neutrophils with maximum amounts of 10^−4^ μmol/10^6^ neutrophils at nanomolar concentrations (half maximal stimulation at ~30 nM) and is toxic at very low concentrations to cultured human epidermal cells (50% loss of colony-forming efficiency at ~3 × 10^−13^ M) [137].

In a method useful for targeting PTX to tumor cells, a monoclonal antibody-enzyme conjugate activating the PTX prodrug *N*-(4'-hydroxyphenylacetyl)palytoxin (NHPAP) was described at the surface of tumor cells. PTX resulted remarkably toxic to H2981 human lung adenocarcinoma, cells, as well as to lymphoma cell lines. NHPAP was 1000 times less toxic than PTX but its cytotoxicity reached that of PTX after combination with penicillin G amidase from *Escherichia coli*. Immunologically specific activation of NHPAP took place after treatment of H2981 cells with the monoclonal antibody conjugate and NHPAP. The authors emphasized that this system is suitable because “the released drug exerts its activity extracellularly, has high potency, and may be able to overcome the multidrug resistant phenotype” [146]. 

The role of different protein kinases (calcium-dependent protein kinase C—PKC, extracellular signal-regulated kinase—ERK 2, c-Jun *N*-terminal protein kinases—JNK, mitogen activated protein kinases—MAPKs, MAPK kinase—MEK) for the activity of PTX on cytosolic calcium concentration and cytoxicity was investigated in primary cultures of cerebellar granule cells [147] observing that the inhibition of ERK 2 and MEK has an effect on PTX cytotoxicity, that at 10 nM is known to induce calcium increase and intracellular acidification [148,149]. In fact, the inhibition of ERK 2 completely inhibits the cytotoxic activity of the toxin, while a partial blockade was observed after MEK inhibition [147]. Recently, in a cytotoxicity research on neuroblastoma cells (Neuro-2a), PTX killed all treated cells at concentrations >10^−9^ M [150].

Head and neck squamous cell carcinoma (HNSCC) cell lines resulted highly sensitive (LD_50_ = 1.5–3.5 ng/mL) to PTX compared to normal epithelial cells. Both the release of LDH and the expression of the sodium/potassium-transporting ATPase subunit alpha1 gene were affected by PTX. The authors supposed a primary activity of PTX on plasma membrane caused loss of cellular integrity [139]. 

The effect of PTX on rat pheochromocytoma PC12 cells has been recently studied. The observed concentration-dependent cytotoxicity was ascribed to the disruption of plasma membrane and to the consequent nonoxidative necrotic damage. Antioxidants or reduced glutathione do not affect this behaviour that in addition does not cause chromatin condensation and DNA fragmentation. PTX seems to cause cell membrane damage through a non-oxidative necrotic process. The exposure to PTX was seen to cause release of lactate dehydrogenase into the culture medium [145]. In Caco-2 cells, PTX at a concentration of 8.9 ± 3.7 × 10^−12^ M reduced the mitochondrial activity by 50% and induced cytotoxicity when tested with Sulforhodamine B assay (EC_50_ = 2.0 ± 0.6 × 10^−11^ M) as well as with LDH release (EC_50_ = 4.5 ± 1.4 × 10^−9^ M) [151].

As concerns other compounds from *Palythoa*, four cell growth inhibitory peptides—palystatins A–D, with relatively low molecular weight (3000–5000)—were isolated from *Palythoa liscia*. Palystatins A–D showed cytotoxic properties against P388 murine lymphocytic leukemia (ED_50_ values = 0.0023 (A), 0.020 (B), 0.0018 (C) and 0.022 (D) μg/mL) [152]. Table 2 shows a summary of data pertinent to papers concerning Hexacorallia.

**Table 2:** Cytotoxicity to different cell lines of compounds extracted from Hexacorallia (Anthozoa).

Species	Compound or material	Cells	Tissue/organ/histology	Organism	IC_50_–ED_50_ (μg/mL)	Ref.
*Epizoanthus* sp.	Alkaloid	HCT8	Colon adenocarcinoma	Human	1.61	[130]
		A549	Lung carcinoma	Human	2.38	
		HT29	Colon adenocarcinoma	Human	0.82	
		P388	Lymphoma	Mouse	1.77	
*Montipora digitata*	Carboxylic acids	P388	Lymphoma	Mouse	5–12	[132]
*Montipora* sp.	Acetylenic compounds	A549	Lung carcinoma	Human	>50	[133]
		SK-OV-3	Ovarian adenocarcinoma	Human	2.5–>50	
		SK-MEL-2	Melanoma	Human	1.4–>50	
		XF498	CNS cancer	Human	1.9–>50	
		HCT15	Colorectal adenocarcinoma	Human	3.7–>50	
*Montipora* sp.	Diacetylenes	A549	Lung carcinoma	Human	3.9->30	[134]
		SK-OV-3	Ovarian adenocarcinoma	Human	1.8–>30	
		SK-MEL-2	Melanoma	Human	1.4–>30	
		XF498	CNS cancer	Human	3.7–>30	
		HCT15	Colorectal adenocarcinoma	Human	3.3–>30	
*Palythoa caribaeorum*	Palytoxin	UKHN-1	Oropharynx squamous cell carcinoma	Human	1.2 (×)	[139]
		UKHN-2	Esophagus squamous cell carcinoma	Human	2.2 (×) approx	
		UKHN-3	Tongue squamous cell carcinoma	Human	3.0 (×)	
*Palythoa liscia*	Palystatins A-D	P388	Lymphoma	Mouse	0.0023–0.02	[152]
*Palythoa tuberculosa*	Palytoxin	H2981	Lung adenocarcinoma	Human	3 × 10^−12^ (+)	[146]
*Commercial source*	Palytoxin	PC12	Pheochromocytoma	Rat	5–8 (♦) approx	[145]
*Tubastrea faulkneri*	Macrolides		Tested on 60 tumour cell lines	Human	0.6–2.5 (^)	[131]

(+) Values expressed as M. (^) Values expressed as μM. (♦) Values expressed as nM. (×) Values expressed as ng/mL.

### 3.3. Cytotoxicity of Extracts from Sea Anemones (Anthozoa)

Reports concerning the cytotoxicity of sea anemones have been published since the late 1970s. *Actinia equina* L. has been widely studied from this point of view. To our knowledge, the first report about the cytotoxicity of *Actinia equina* venom was published in 1976 [153], when a potent cytotoxic activity of Equinatoxin was observed by dye exclusion test on Ehrlich carcinoma and L1210 leukaemia inoculated in swiss albino mice with ED_50_s of a few ng/mL. The authors observed that concentrations higher than ED_50_ produced extensive cell lysis and the cytotoxic effects of Equinatoxin were inhibited by phospholipids; on this basis, they suggested that the mechanism of action “may be related to interactions with lipids or other charged components of cell membrane”. The cytotoxic and cytolytic effects of Equinatoxin II (EqT II), as well as its concentration-dependent cytocidal and cytostatic effects, were subsequently emphasized on V-79-379 A cells [154]. On the basis of results showing that after treatment with EqT II pituitary glands bovine lactotrophs suffered a rapid rise in cytosolic Ca^2+^ activity and that Ca^2+^ permeable ion channels are produced after incorporation of EqT II into planar lipid bilayers, Zorec *et al.* [155] suggested the cytotoxicity of EqT II can be ascribed to the formation of cation (Ca^2+^) permeable channels in cell membranes. Subsequenty, EqT II was observed to produce pores affecting the permeability of V79-379 A cells plasmalemma for metabolites and ions. Treated cells were killed by approximately 75 μg/10^6^ cells EqT II and the toxic effects were lowered by serum. Less than 37.5 μg EqT II/10^6^ cells did not produce significant change in cell membrane fluidity [156]. In another study, crude extracts from nematocyst and surrounding tissues of the sea-anemone *Actinia equina* were tested on V79 fibroblasts observing that 150,000 nematocysts/mL caused a decrease of cell survival up to approximately 60%–70% according to the utilized method of evaluation (Trypan blue dye exclusion and neutral red assay, respectively) after one hour treatment and killed all treated cells after two hours. At doses ranging from 150,000 to 15,000 nematocysts/mL, the venom was not genotoxic [157]. Considering that the pore forming toxin EqT II damages cells through the activity of an amphiphilic *N*-terminal α-helix on membrane, and considering also that a “normally active *N*-terminal mutant, containing one single cys in the amphiphilic α-helix, becomes totally inactive when it is bound to avidin via a biotinylated linker”, in a interesting study Potrich *et al.* [158] chose a peptide containing a tumour protease cleavage site as a linker, making an enzymatically activable conjugate selective for tumour cells that was seen to be activated *in vitro* by cathepsin B and metalloproteinases, known to be involved in cancer progression and tumour migration [159,160]. The conjugate was partly activated by ZR 751 (human breast carcinoma), MCF 7 (human breast adenocarcinoma) and HT 1080 (human fibrosarcoma) cells, but was inactive on human red blood cells used as controls. The cytotoxicity was dependent on the expressed amount of cathepsin B activity. MCF 7 cells, expressing the highest enzymic activity, were found to be the most sensitive tumour cells; thus, these tumour cells could be killed by a conjugate of EqT II specifically activated by tumor proteases [158].

EqTx-II from *Actinia equina* was studied for cytotoxicity against human glioblastoma U87 and A172 cell lines at concentrations ranging from 0.001 to 10 mg/mL. After 24 h of treatment, 10 mg/mL EqTx-II was found to be remarkably cytotoxic and reduced the viability of U87 and A172 cells to 60% and 48%, respectively, but was not significantly toxic for normal cells (10 mg/mL EqTx-II decreased viability to 80%). Noncytotoxic concentrations of EqTx-II (0.3 mg/mL) were seen to improve the cytotoxicity of chemotherapeutics cytosine arabinoside, doxorubicin, and vincristine utilized at low concentrations. As these chemotherapeutics are known to be highly cytotoxic and to induce adverse effects, the utilization of sea anemone toxins could allow a reduction of the therapeutic dosage [161]. EqTx-II was found to decrease cell viability of U87 glioblastoma cells through a necrosis-like mechanism and increased lactate dehydrogenase (LDH) release in a concentration-dependent manner. It is also able to activate intracellular signaling pathways. Pre-treatment with inhibitors of mitogen-activated/extracellular regulated kinase (MEK1), protein kinase C (PKC) or Ca^2+^/calmodulin-dependent kinase II (CaMKII) prevents EqTx-II toxicity. This allowed the authors to suggest that calcium entry, activation of MEK1, PKC and CaMKII pathways are involved in the cytotoxicity induced by pore-forming toxins [162]. A recent article showed that non-cytotoxic concentrations of EqTx-II seems to fight the cytotoxicity induced by chemotherapeutics temozolomide (TMZ) and etoposide (VP-16) on glioblastoma cells *in vivo* and *in vitro* thus reducing the adverse effects of these drugs [163]. 

Other Mediterranean anemones were found to be cytotoxic: crude venom from sea-anemone *Anemonia sulcata* produced growth inhibition and rapid detachment of V79 cells; in particular, cells treated with the highest tested dose (150,000 nematocysts/mL) died within one day of treatment, and those treated with 30,000 nematocysts/mL died within two days [164,165]. Short-time tests (3 h treatment) on V79 cells gave an IC_50_ value of 65.0 × 10^3^ nematocysts/mL [166]. Crude nematocystic extracts (0.6 nematocysts/μL) from the anthozoan *Aiptasia mutabilis* were found to be highly cytotoxic inducing significant cellular necrosis on renal monkey Vero cells, with an IC_50_ of approximately 2 nematocysts/μL, and on human epithelial HEp-2 cells. The venom was found to be inactivated by moderate heat, conservation at low temperature or freezing, and by non-neutral pH values. Two main cytolytic components with molecular masses of 95 and 31 kDa, respectively, were identified [167]. Therefore, in spite of the scarce toxicity *in vivo* of Mediterranean Cnidaria [168]), a strong cytotoxic activity *in vitro* was demonstrated for some Mediterranean sea-anemones.

As concerns other sea anemones, the crude extract from the *Urticina piscivora* and the basic protein UpI isolated from crude extract were investigated on oral human epidermoid carcinoma cells KB (ATCC CCL 17), mouse lymphocyte leukemia cells L1210, and human embryonic lung diploid cells HEL 299; the crude extract was more cytotoxic than the isolated protein [25]. Src I from the sea anemone *Sagartia rosea* was found to be cytotoxic for cultured NIH/3T3 (Swiss mouse embryo), U251 (glioblastoma), NSCLC (non-small cell lung carcinoma), BEL-7402 (liver carcinoma), and BGC-823 (stomach adenocarcinoma) cells, depending on toxin concentration and incubation time. IC_50_ values in the absence of serum ranged from 2.8 to 7.4 μg/m; NSCLC cells were the most sensitive. The presence of serum partially inhibited the cytotoxicity of Src I [26]. Fedorov *et al.* [169] studied the RTX-A actinoporin isolated from the tropical sea anemone *Heteractis crispa* (*Radianthus macrodactylus*) and discovered that it can prevent the malignant transformation of mouse epidermal JB6 P^+^ Cl41 cells with a useful INCC_50_ (inhibition of number of colonies formed in soft agar C_50_) value (0.034 nM), 17 times lower than the cytotoxic concentration (IC_50_ = 0.57 nM). RTX-A was demonstrated to be cytotoxic also to human cancer cell lines, including promyelocytic leukemia cells (HL-60; IC_50_ = 1.06 nM), breast cancer cells (MDA-MB-231; IC_50_ = 4.64 nM), cervix carcinoma cells (HeLa; IC_50_ = 2.26 nM), monocytic leukemia cells (THP-1; IC_50_ = 1.11 nM) and colon cancer cells (SNU-C4; IC_50_ = 4.66 nM), as well as to induce dose-dependent apoptosis in JB6 P^+^ Cl41 cells. The effects of RTX-A on the activity of proteins (AP-1, NF-κB) activating the expression of genes involved in tumor progression [170,171] were also studied in JB6 P^+^ Cl41 cells showing the time- and dose-dependent inhibition (10%–60%) of basal AP-1- and NF-κB-dependent transcriptional activity at concentrations 0.1–1.6 nM. At this concentration, RTX-A was shown to induce apoptosis with a p53-independent mechanism, suppressing time- and dose-dependently the p53-dependent transcriptional activity of JB6 P^+^ Cl41 cells [169]. The pore-forming cytolytic toxins (Bc2) produced by *Bunodosoma caissarum* was investigated for cytotoxicity against human glioblastoma U87 and A172 cell lines at concentrations ranging from 0.001 to 1 mg/mL. After 24 h of treatment, <0.1 mg/mL Bc2 did not exert cytotoxicity but 1 mg/mL reduced significantly U87 and A172 cell viability (50% and 65%, respectively). As already seen for EqTx-II, noncytotoxic concentrations of Bc2 (0.1 mg/mL) were seen to improve cytotoxicity of the chemotherapeutics cytosine arabinoside, doxorubicin, and vincristine, utilized at low concentrations, on GBM cells. Therefore, the utilization of Bc2 could allow a reduction 10–300-fold the dosage of these chemotherapeutics that are known to be highly cytotoxic and to induce adverse effects. Bc2 did not significantly reduce the viability of normal rat astrocytes [161]. Similar results were recently obtained stating that sea anemones produce compounds with pharmacological activities that may be useful to increase cisplatin efficacy. The expositions to 50 μg/mL crude venom from the sea anemone *Bunodeopsis globulifera* and to 25 μg/mL and 50 μg/ mL, respectively, of two derived fractions (F1 and F2) were seen to increase cisplatin cytotoxicity to human lung adenocarcinoma cells inducing a reduction in cell viability of approximately 50%. From these results, the authors conclude that “the combination of antineoplastic drugs and sea anemone toxins might allow a reduction of chemotherapeutic doses and thus mitigate side effects” [172]. Table 3 shows a summary of data pertinent to papers concerning sea anemones.

**Table 3:** Cytotoxicity to different cell lines of compounds extracted from sea anemones (Anthozoa).

Species	Compound or material	Cells	Tissue/organ/histology	Organism	IC_50_–ED_50_ (μg/mL)	Ref.
*Bunodosoma caissarum*	Bc2	U87	Glioblastoma	Human	Not indicated	[161]
		A172	Glioblastoma	Human		
*Actinia equina*	Equinatoxin II	V-79-379 A	Normal lung fibroblasts	Chinese hamster	8.8 × 10^−10^ (*)	[154]
*Actinia equina*	Crude venom	V79	Normal lung fibroblasts	Chinese hamster	87.9 × 10^3^ (**)	[157]
*Actinia equina*	Equinatoxin II-I18C mutant	MCF 7	Breast adenocarcinoma	Human	0.2–0.3	[158]
		ZR 751	Breast carcinoma	Human	5.8	
		HT 1080	Fibrosarcoma	Human	14.2	
*Actinia equina*	EqTx-II	U87	Glioblastoma	Human	Not indicated	[161]
		A172	Glioblastoma	Human		
*Aiptasia mutabilis*	Crude venom	Vero	Normal kidney cells	Monkey	2000 (**)	[167]
		Hep-2	Epithelial carcinoma	Human	Not indicated	
*Anemonia sulcata*	Crude venom	V79	Normal lung fibroblasts	Chinese hamster	65.0 × 10^3^ (**)	[166]
*Heteractis crispa*	Actinoporin RTX-A	HL-60	Promyelocytic leukemia	Human	1.06 (♦)	[169]
		HeLa	Cervix carcinoma	Human	2.26 (♦)	
		THP-1	Monocytic leukemia	Human	1.11 (♦)	
		MDA-MB-231	Breast cancer	Human	4.64 (♦)	
		SNU-C4	Colon cancer	Human	4.66 (♦)	
		Cl 41	Epidermal cells	Mouse	0.57 (♦)	
*Sagartia rosea*	Acidic actinoporin Src I	U251	Glioblastoma	Human	3.5	[26]
		NSCLC	Non-small cell lung carcinoma	Human	2.8	
		BEL-7402	Liver carcinoma	Human	3.6	
				Human	7.4	
		BGC-823	Stomach adenocarcinoma	Mouse	3.4	
		NIH/3T3	NIH Swiss embryo			
*Urticina piscivora*	Crude extract	KB	Epidermoid carcinoma (#)	Human	6.54	[25]
		HEL299	Embryonic lung	Human	10.07	
		L1210	Lymphocytic leukemia	Mouse	2.34	
*Urticina piscivora*	UpI (protein)	KB	Epidermoid carcinoma (#)	Human	40.32	[25]
		HEL299	Embryonic lung	Human	29.99	
		L1210	Lymphocytic leukemia	Mouse	29.74	

(*) Values expressed as mole/L; (**) values expressed as nematocysts/mL; (♦) values expressed as nM; (#) to date considered a HeLa cell contaminant.

### 3.4. Cytotoxicity of Extracts from Scyphozoa

Jellyfish venoms have been studied since the early 1980s on cultured cells. High doses of crude or fractionated venom from sea nettle *Chrysaora quinquecirrha* (Semaeostomeae) were seen to produce morphological changes in CHO K-1 cells, to inhibit cell growth and to interfere with intracellular uridine and thymidine incorporation, while low doses induced mitogenic activity; furthermore, rabbit erythrocytes were agglutinated by partially purified venom in the presence of calcium [173]. A contemporary study showed that venom from *Chrysaora quinquecirrha* induced nuclear alterations on K-1 cells (Chinese hamster ovary) with production of multi-nucleated cells and multiple nucleoli as well as loss of peripheral chromatin and dissolution of intercellular collagen as a consequence of its enzymatic (collagenase, protease and lectin-like) activity [174]. Cao *et al.* [175] observed that the venom of *Chrysaora quinquecirrha*, which is composed of several polypeptides, is highly toxic for non-malignant human hepatocytes ATCC CCL-13. A transient increase and a subsequent decrease of metabolic activity, evaluated as production of acidic metabolites, followed by cell death was observed within 30 min caused by 28 μg protein/mL medium. Higher doses showed the same trend but delayed activity. On the contrary, the lowest studied dose (0.3 mg protein/mL medium) caused an increase of metabolic activity that did not fall within 2 h. Phosphorylation or alkylation of cell protein(s) was observed to interfere with the toxicity of sea nettle venom [175]. The venom of *Chrysaora quinquecirrha* was observed to also be toxic to cultured rat hepatocytes, but its activity was not Ca^2+^ dependent [176]. 

The jellyfish *Cyanea capillata* and *Cyanea lamarckii* (*Semaeostomeae*) were the subject of a study aimed to evaluate the enzymatic, cytotoxic and hemolytic potency of their venoms. Purified cnidocyst extracts from fishing and mesenteric tentacles of both jellyfish induced strong damage to hepatoma cells HepG2 (10% cell survival) after treatment with 33.3 μg_protein_/mL. PLA2-like activity was demonstrated in extracts from mesenteric and fishing tentacles of both jellyfish [46]. Cultured rainbow trout gill cells RTgill-W1 exposed to increasing protein concentrations of crude venoms extracted from fishing tentacles and oral arms of *Cyanea capillata* and *Aurelia aurita* showed dose-dependent survival decrease evidenced by detachment, clumping and lysis and morphological changes after 1 h of treatment. The cytotoxic effect was evident starting from a concentration >2.0 μg/mL of venom (protein). *C. capillata* oral arms venom induced a percent cell viability ranging from 7.4 to 36.4 according to the size of the umbrella. The treatment with 0.2 μg 10^4^ cells^−1^ oral arms and fishing tentacles venom from *A. aurita* allowed 15% and 18% cell survival, respectively. In this research, the venom from oral arm cnidocysts was indicated to be more cytotoxic that that from fishing tentacles at the same protein concentration [177]. A novel cytotoxic protein was isolated from the fishing tentacle venom of *Cyanea capillata* and tested on human hepatocytes HepG2. The purified protein CcTX-1 (MW of the main isoform = 31.17 kDa) showed strong cytotoxicity and caused the death of nearly all treated cells at a concentration of 1.3 μg/mL. The amino acid sequence of CcTX-1 was shown to be similar to that of haemolysins from Cubozoans *Carybdea alata* (CaTX-1) and *Carybdea rastonii* (CrTX-1) [4]. In another paper, the size of fishing tentacles and oral arms as well as the nematocyst (A-isorhizas and O-isorhizas) number and size that correspond to the size of the umbrella, were correlated with the cytotoxic and neurotoxic potency in differently-sized *Cyanea capillata* (L.) showing that the greater the specimen, the higher the cytotoxicity and neurotoxicity. Rainbow trout (RTgill-W1) cells were highly sensitive to the crude venom with a minimal effect at concentrations of 1 μg/mL and 2 μg/mL for venoms from oral arms and from fishing tentacles, respectively, and EC_50_ values ranging from 3.9–10.1 μg/mL for small medusae and from 8.0 to 4.5 μg/mL for a large medusa. All samples, both from large and from small medusae, showed a dose-dependent neurotoxic activity i*n vitro* on mouse neuroblastoma Neuro 2A CCL 131 cell line, with a remarkable neurotoxic activity of fishing tentacle venom from the large medusa [47]. The venom from the nematocysts of *Cyanea nozakii* Kishinouye was assessed for cytotoxicity on Bel-7402 and SMMC-7721 human hepatoma cells and on H630 human colon cancer cells. H630 cells showed the highest sensitivity (IC_50_ = 15.9, 8.8 and 5.1 μg/mL after incubation for 12, 24 and 48 h, respectively) followed by Bel-7402 (17.9 μg/mL) and SMMC-7721 (24.3 μg/mL). The cytotoxicity was time- and dose-dependent, showed the best efficacy at pH values ranging from 4.5 to 8.5, was affected by temperature and lost its efficacy when pre-incubated at temperatures of 60 and 80 °C. The venom seems to be able to damage cell membrane as verified with the percent Lactate dehydrogenase (LDH) release which increased with time and venom concentration [178].

The mauve stinger *Pelagia noctiluca* is the main stinging Mediterranean jellyfish, able to cause remarkable toxicity and systemic damage [168,179]. The first evidence of cytotoxicity induced by crude venom of this jellyfish was reported in mid-1990s when long-term treatment with the crude venom (150,000 nematocysts/mL) caused cell growth decrease (38%) of V79 cells in comparison to controls. Lower doses (30,000 and 15,000 nematocysts/mL) caused cell growth decreases of 45% and 61%, respectively [165]. Subsequently, the crude venom of this jellyfish was seen to induce ATP increase in treated V79 cells and remarkable survival decrease of 1–3 hour-treated cells with IC_50_ values ranging from 74.2 × 10^3^ to 29.8 × 10^3^ nematocysts/mL [180]. Recently, *Pelagia noctiluca* venom was shown to cause overproduction of ROS with oxidative damage, increasing catalase activity and induction of lipid peroxidation (increasing malondialdehyde generation) with consequent genotoxic effects and DNA fragmentation in human colon (HCT 116) cancer cells [181]. Morabito *et al.* [2] studied the effect of nematocyst crude venom from *Pelagia noctiluca* on neuronal-like cells derived from human neuroblastoma SH-SY5Y. Doses ranging from 0.05 to 0.5 μg/mL of lyophilized and re-suspended in PBS crude venom were shown to induce oxidative stress and to affect cell viability through a dose- and time-dependent production of intracellular reactive oxygen species (ROS) and changes in mitochondrial transmembrane potential. The antioxidant *N*-acetyl-cysteine was able to counteract cell viability decrease and ROS production. The induction of oxidative stress was supposed to be caused by disruption of mitochondrial membrane with the consequence to inhibit mitochondrial respiration and uncouple oxidative phosphorylation [2]. 

The anti-tumoral activity of crude venom and of four crude venom fractions (F1, F2, F3, F4) extracted from *Pelagia noctiluca* through sephadex G-75 chromatography was investigated for anti-proliferative and anti-cell adhesion of human glioblastoma (U87) cells. The cytotoxicity of unfractionated crude venom and of two fractions (F1 and F3) was emphasized (IC_50_ values of 180, 125 and 179 μg/mL, respectively). An important time-dependent, anti-proliferative activity was observed in treatments with F1 and F2 while less evidence was shown by crude venom (fair activity) and F3 (low activity). The crude venom and F1, F2 and F3 fractions also caused a dose-dependent inhibition of cell adhesion to fibrinogen. The authors attributed this activity to the interaction of venom with integrins [1] and stated that *Pelagia noctiluca* venom may play a role in the development of anti-cancer drugs. Very recent results indicate that the toxicity of *Pelagia noctiluca* seems to be caused by the generation of reactive oxygen species (ROS). In fact, the dose- and time-dependent mortality observed on Vero cells (green monkey kidney) after treatment with crude extracts from *P. noctiluca* nematocysts was lowered by Vitamin E, which therefore seems to have a cytoprotective effect against the oxidative stress that was supposed to be the major mechanism of toxicity of *Pelagia noctiluca* [182].

As concerns the Rhizostomeae, the crude toxin from *Rhizostoma pulmo* was found to produce cytotoxic effects and growth inhibition on V79 cells both after short- and long-time treatments with an IC_50_ value of 39.9 × 10^3^ nematocysts/mL [43,165,166]. The crude venom from *Rhizostoma pulmo* was analyzed by HPLC after soaking of oral arms in distilled water, obtaining a sample devoid of nematocysts. HPLC analysis provided five peaks and the subsequent preliminary cytotoxic assays on V79 cells using the single HPLC fractions indicated that a fraction, named fraction “b”, was highly cytotoxic [44]. The cytotoxicity of collagen from *Rhizostoma pulmo* and the effects on cell adhesion of primary fibroblasts, osteoblastic (MG-63), epithelial (HaCat) and fibrosarcoma (HT-1080) cell lines cultured on jellyfish collagen-coated wells was studied recently. After two and eight days, the amount of viable cells was not significantly different from controls indicating the harmlessness of jellyfish collagen [183].

*Cotylorhiza tuberculata* was always considered a non-dangerous jellyfish. In spite of this very recent result, [3] indicate that fractionated extracts of whole jellyfish characterized by HPLC, GC-MS and SDS-PAGE showed antioxidant activity and affected cell viability and intercellular communication of breast cancer cells (MCF-7) and human epidermal keratinocytes (HEKa). MCF-7 cells were found to be more sensitive to the extracts. The modulation of intercellular communication was hypothesized to play a role in the anticancer bioactivity [3]. 

The incidence and growth of SNC tumors induced by *N*-Ethyl-*N*-Nitrosourea were shown to be affected by the crude venom of *Cassiopea xamachana* [184]. Crude venom from the giant jellyfish *Nemopilema nomurai* (Rhizostomeae) occurring in the waters of China, Korea and Japan was found to be cytotoxic and hemolytic *in vitro* and showed high cytotoxicity against H9C2 heart myoblasts (LC_50_ = 2 μg/mL). These findings allowed the authors to assert that *Nemopilema nomurai* venom can exert a selective toxicity on cardiac tissue *in vivo*. Venom activity was retained at low temperature (≤20 °C) and high pH (pH ≤ 12), but was lost at high temperature (≥60 °C) and at low pH (pH ≤ 4) [45]. 

Cell-damaging activity of other scyphozoans has been related with the proteolytic activity of their venoms. Assessing the cytotoxicity of four Scyphozoan jellyfish (*Nemopilema nomurai*, *Rhopilema esculenta*, *Cyanea nozakii*, and *Aurelia aurita*) on NIH 3T3 cells, a cytotoxic potency scale *C. nozakii* > *N. nomurai* > *A. aurita* > *R. esculenta* was reported. The metalloproteinases were indicated to play an important role in jellyfish toxicity [185]. A very recent paper [22] evaluated the activity of extracts from Coronatae scyphozoans *Atolla vanhoeffeni*, *Atolla wyvillei* and *Periphylla periphylla* on mouse leukemia L1210. Only for *Atolla vanhoeffeni* was it possible to calculate the IC_50_ value which was found to be very high (740 mg/mL). Table 4 shows a summary of data pertinent to papers concerning Scyphozoa.

**Table 4:** Cytotoxicity to different cell lines of compounds extracted from jellyfish (Scyphozoa).

Species	Compound or material	Cells	Tissue/organ/histology	Organism	IC_50_–ED_50_ (μg/mL)	Ref.
*Atolla vanhoeffeni*	Water soluble extract	L1210	Lymphocytic leukemia	Mouse	740 (°)	[22]
*Cyanea capillata*	Fishing tentacle extract	HepG2	Hepatoma	Human	20.3	[46]
*Cyanea capillata*	Preparations from nematocyst suspensions	RTgill W1 Neuro 2A	Normal gill Neuroblastoma	Rainbow trout Mouse	3.9–10.1	[47]
					Not indicated	
*Cyanea nozakii*	Crude extract	Bel-7402	Hepatoma	Human	17.9	[178]
		SMMC-7721	Hepatoma	Human	24.3	
		H630	Colon cancer	Human	5.1–15.9	
*Pelagia noctiluca*	Crude venom	V79	Normal lung fibroblasts	Chinese hamster	29.8–74.2 × 10^3^ (**)	[180]
*Pelagia noctiluca*	Crude venom	HCT 116	Colon cancer	Human	320	[181]
*Pelagia noctiluca*	Crude venom	U87	Glioblastoma	Human	180	[1]
*Pelagia noctiluca*	Crude venom	Vero	Normal kidney cells	Monkey	64–112 × 10^3^ (**) (MTT) 20–90 × 10^3^ (**) (NR)	[182]
*Rhizostoma pulmo*	Crude venom	V79	Normal lung fibroblasts	Chinese hamster	39.9 × 10^3^ (**)	[43,165,166]
*Cotylorhiza tuberculata*	Extract (pigments, fatty acids, polypeptides)	MCF-7	Breast adenocarcinoma	Human	0.015	[3]
		HEKa	Normal keratinocytes	Human		
*Nemopilema nomurai*	Crude venom	H9C2	Heart myoblasts	Rat	2.0 (*)	[45]
		C2C12	Muscle myoblasts	Mouse	12.2 (*)	

(*) Values expressed as LC_50_; (**) values expressed as nematocysts/mL; (°) as reported in the paper in the legend of Table 1.

### 3.5. Cytotoxicity of Extracts from Hydrozoa

Reports of the cytotoxic properties of extracts from hydrozoans are quite scarce; in early 1980s, a study showed that venom from Portugese man-o’war *Physalia physalis* induced nuclear alterations on K-1 cells (Chinese hamster ovary) with production of multi-nucleated cells and multiple nucleoli, as well as loss of peripheral chromatin and dissolution of intercellular collagen, as a consequence of the enzymatic activity (collagenase, protease and lectin-like) of venom [174]. Subsequently, the separation of nematocysts of *Physalia physalis* on the basis of size emphasized that venom from small nematocysts (10.6 nm diameter) was lethal to *in vitro* cultured chick embryonic cardiocytes at 0.6 μg protein/culture; on the contrary, the venom from great nematocysts (23.5 nm diameter) at 20 μg protein/culture was ineffective [186]. Recently, the venom of *Physalia physalis* was tested on mouse fibroblast cell line L-929 [187], and two novel toxins (PpV9.4, PpV19.3, with molecular weights 550.7 and 4720.9 Da, respectively) were purified from this siphonophore. Pancreatic beta-cells cultured for one day in RPMI-1640 increased insulin secretion and showed cytosolic Ca^2+^ increase after treatment with both toxins [188]. The evaluation of cytotoxicity to V79 cells of crude venom from *Aequorea aequorea*, a species that was never studied before from the toxicological point of view, indicated an IC_50_ value of 76.6 × 10^3^ nematocysts/mL [166] and a remarkable activity after long-time treatment [165]; therefore, this jellyfish seems to be able to affect cell growth rate with slow activity in time. A noncnidocystic toxin, homologous with pore-forming proteins having specific activity toward arthropods, was isolated in green hydra *Chlorohydra viridissima* [189]. Its toxicity was seen to be differential toward insect (SF-9) and human (HEK293) cultured cells. Large amounts of this depolarizing toxin found in *Hydra* could be secreted into the coelenteric fluid and have a role in keeping the prey paralyzed after ingestion perhaps allowing also an extracellular digestion [189]. Kawabata *et al.* [22] recently evaluated on mouse leukemia L1210 cells the activity of extracts from deep-sea hydrozoan jellyfish, the anthomedusan *Pandea rubra*, the trachymedusae *Arctapodema* sp., *Colobonema sericeum*, *Crossota rufobrunnea*, *Halicreas minimum*, *Pantachogon haeckeli*, and the narcomedusae *Aeginura grimaldii*, *Aegina citrea*, *Solmissus* sp. A clear cytotoxicity was caused especially by *Aegina citrea* (IC_50_ = 100 μg/mL), *Pantachogon haeckeli* (IC_50_ = 160 μg/mL), *Aeginura grimaldii* (IC_50_ = 170 μg/mL), and *Arctapodema* sp. (IC_50_ = 190 μg/mL) extracts; higher values were obtained with *Colobonema sericeum* (IC_50_ = 420 μg/mL) and *Halicreas minimum* (IC_50_ = 750 μg/mL) extracts. As heat- and methanol-treated extracts did not show bioactivity, a high incidence of water-soluble bioactive substances and the presence of unstable bioactive proteins in water-soluble extracts were supposed to occur in these hydrozoans [22].

### 3.6. Cytotoxicity of Extracts from Cubozoa

Cubozoans are well known for their danger and for the potential lethal effects that they can induce on humans. In spite of this, the research on the activity of cubozoan venoms at cellular level is still scarcely developed. Sun *et al.* [190] demonstrated that the growth of human U251 and rat C6 malignant glioma cells and that of transformed vascular endothelial ECV 304 cells was inhibited by box jellyfish *Chiropsalmus quadrigatus* venom that caused also DNA fragmentation and signs of apoptosis. In U251 cells, the increase of p53 expression was recorded. This was indicated as one of mechanisms through which the venom of *Chiropsalmus quadrigatus* induces apoptosis in glioma and endotelial cells, thus the possible application of apoptosis-inducing venom in the therapy of gliomas has been emphasized. The venom from the box jellyfish *Chironex fleckeri* specimens collected in different zones of Northern Australia was screened for cytotoxicity on rat aortic smooth muscle cell line A7r5. A concentration-dependent cytotoxicity (IC_50_ values ranging from 0.7 to 0.03 μg/mL) was observed but this activity differed according to the season in which the specimens were collected. These differences were observed also in the composition of venom that was analyzed by size exclusion HPLC and SDS-PAGE profiles. The authors concluded that “there is considerable geographical variation in the composition of *C. fleckeri* venoms which [...] may explain the geographical variation in reported deaths” [191]. The unfractionated (whole) venom from *C. fleckeri* was see to cause dose-response detachment of human cardiac myocytes from culture wells. After fractionation of the venom with size exclusion chromatography (FPLC), one portion of the venom (mean size approximately 65 kDa) was seen to cause approximately 80% cell detachment from substratum and death after 30 min [192].

## 4. Conclusions

Natural venoms produced by cnidarians—as a rule utilized for defense/offense purposes and for predation—are a research subject of great concern because many of these compounds can have pharmacological activity and could be used as future drugs. As a matter of fact, it is well known that approximately one third of the best selling drugs are derived from natural sources or have been developed on the basis of lead natural structures [193]. For this reason, the research of novel drugs is focused on the discovery of new compounds among which those derived from marine organisms are viewed with particular interest. The cytotoxicity of cnidarian venoms has been studied for decades leading to results that emphasize the strong activity of several of them, in particular against cancer cells, and the literature on this subject is enormous. Several cnidarian venoms have been demostrated to inhibit cell growth—for example, interfering with cell metabolism or inhibing DNA synthesis—and some of them were seen to be able to block the cell cycle. Furthermore, because some cnidarian venoms were indicated to be apoptosis-inducing, this could be another tool to be used against target malignant cells. Several cnidarian venoms were found to be aspecifically cytotoxic on different cancer or normal cells but some of them showed specificity for definite cell lines. On the whole, many types of venom were found to be active on leukemic cells and colorectal adenocarcinoma cells. In addition, the importance of some compounds extracted from cnidarians is a matter of concern because they seem to improve the activity of chemotherapeutic drugs at appropriate venom concentration. This could allow a reduction in the amount of administered chemotherapeutics reducing also their unpleasant effects. In conclusion, cnidarian venoms, due to some remarkable features such as their capability to produce damage at cellular and tissue levels, to affect the permeability of cell membrane and ion exchange, to cause cell lysis or to modify metabolic pathways, could be utilized as weapons against specific targets or for different therapeutic purposes. Therefore, further research about these compounds is essential to elucidate their action mechanisms and to make them useful for therapy. 
